# Two plant membrane‐shaping reticulon‐like proteins play contrasting complex roles in turnip mosaic virus infection

**DOI:** 10.1111/mpp.70017

**Published:** 2024-10-16

**Authors:** Guanwei Wu, Liping Wang, Rongrong He, Xiaoyan Cui, Xin Chen, Aiming Wang

**Affiliations:** ^1^ London Research and Development Centre, Agriculture and Agri‐Food Canada London Ontario Canada; ^2^ Jiangsu Key Laboratory for Horticultural Crop Genetic Improvement, Institute of Industrial Crops Jiangsu Academy of Agricultural Sciences Nanjing China; ^3^ State Key Laboratory for Managing Biotic and Chemical Threats to the Quality and Safety of Agro‐Products, Institute of Plant Virology Ningbo University Ningbo China; ^4^ State Key Laboratory of Agricultural Microbiology Huazhong Agricultural University Wuhan China; ^5^ Department of Biology Western University London Ontario Canada

**Keywords:** antiviral factor, cell‐to‐cell movement, *Potyvirus*, proviral factor, reticulon, virus replication complex

## Abstract

Positive‐sense RNA viruses remodel cellular cytoplasmic membranes as the membranous sources for the formation of viral replication organelles (VROs) for viral genome replication. In plants, they traffic through plasmodesmata (PD), plasma membrane‐lined pores enabling cytoplasmic connections between cells for intercellular movement and systemic infection. In this study, we employed turnip mosaic virus (TuMV), a plant RNA virus to investigate the involvement of RTNLB3 and RTNLB6, two ER (endoplasmic reticulum) membrane‐bending, PD‐located reticulon‐like (RTNL) non‐metazoan group B proteins (RTNLBs) in viral infection. We show that RTNLB3 interacts with TuMV 6K2 integral membrane protein and RTNLB6 binds to TuMV coat protein (CP). Knockdown of RTNLB3 promoted viral infection, whereas downregulation of RTNLB6 restricted viral infection, suggesting that these two RTNLs play contrasting roles in TuMV infection. We further demonstrate that RTNLB3 targets the α‐helix motif ^42^LRKSM^46^ of 6K2 to interrupt 6K2 self‐interactions and compromise 6K2‐induced VRO formation. Moreover, overexpression of AtRTNLB3 apparently promoted the selective degradation of the ER and ER‐associated protein calnexin, but not 6K2. Intriguingly, mutation of the α‐helix motif of 6K2 that is required for induction of VROs severely affected 6K2 stability and abolished TuMV infection. Thus, RTNLB3 attenuates TuMV replication, probably through the suppression of 6K2 function. We also show that RTNLB6 promotes viral intercellular movement but does not affect viral replication. Therefore, the proviral role of RTNLB6 is probably by enhancing viral cell‐to‐cell trafficking. Taken together, our data demonstrate that RTNL family proteins may play diverse complex, even opposite, roles in viral infection in plants.

## Graphical abstract

Plant membrane‐shaping reticulon‐like protein RTNLB3 inhibits turnip mosaic virus infection by suppression of viral replication, whereas RTNLB6 is proviral by enhancing viral cell‐to‐cell movement.

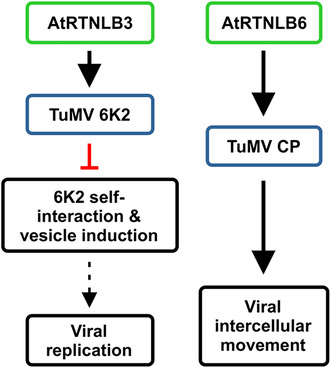

## 1 INTRODUCTION

Reticulon homology proteins (RHPs), including reticulons (RTNs) discovered in vertebrates and RTN‐like protein (RTNLs) in other eukaryotes, are a class of membrane‐bound proteins in the eukaryotic kingdom (Yang & Strittmatter, 2007). Plant RTNLs belong to the non‐metazoan group B protein family (RTNLBs). RHPs contain a carboxy‐terminal reticulon homology domain (RHD) that comprises two characteristic large hydrophobic regions flanking a hydrophilic loop (Zhang & Hu, 2016). Each of these two hydrophobic regions is further composed of two transmembrane domains (TMDs), which form a W‐like topology (Voeltz et al., 2006). RHDs play essential functions in diverse biological processes and are predominantly associated with the endoplasmic reticulum (ER) to help shape the ER membrane into tubules via induction and maintenance of high membrane curvature (D'Eletto et al., 2020). This curvature‐generating and ‐stabilizing function is attributed to the TMDs in the RHD (Zurek et al., 2011). Overexpression of RTNLs in plants may induce severe constrictions of ER tubules and conversion of ER membrane sheets into tubules (Sparkes et al., 2010; Tolley et al., 2008), whereas overexpression or downregulation of the RTNLs in yeast causes morphological alterations of the tubular ER (De Craene et al., 2006; Voeltz et al., 2006).

The majority of known plant‐infecting viruses have a small, single‐stranded positive‐sense (+ss) RNA genome. A common feature of infection by +ssRNA viruses is the formation of the viral replication complexes or organelles (VRCs or VROs) for viral genome replication (He et al., 2023). It is well established that after entry into host cells, +ssRNA viruses target and remodel preferred organelles of the cellular endomembrane system for VRO biosynthesis. Due to their very limited coding capacity, +ssRNA viruses highjack a number of host factors such as RHPs to accomplish this process (Diaz & Ahlquist, 2012; Hyodo & Okuno, 2016; Wang, 2015). The requirement of RHPs for +ssRNA virus replication was first demonstrated in human cells. In this seminal work, Tang et al. (2007) demonstrated that human RTN3 interacts with the viral RNA and membrane‐binding 2C protein of enterovirus 71 (EV71) and RNA interference (RNAi)‐mediated downregulation of RTN3 supresses EV71 replication. Reintroduction of non‐degradable RTN3 into the knockdown cells rescues EV71 replication and infectivity. Moreover, the 2C of other enteroviruses such as poliovirus and coxsackievirus A16 in the *Picornaviridae* family is also an interactor of RTN3, implying RTN3 may be a common host factor for enteroviral infection (Tang et al., 2007). Consistent with these findings, RTN3.1A was shown to play a crucial role in the remodelling of host membranes for efficient replication by three flaviviruses, West Nile virus (WNV), dengue virus (DENV) and Zika virus (ZIKV) (Aktepe et al., 2017). RTN3.1A stabilizes viral proteins within the ER by interacting with the viral NS4A protein to promote viral replication. However, in some other studies, RTN3 seems to function as a restriction factor. For example, RTN3 was found to suppress hepatitis C virus (HCV) replication by disrupting the NS4B self‐interaction (Wu et al., 2014). An RHD‐containing FAM134 family protein inhibits WNV and DENV infection by facilitating autophagy‐mediated ER degradation (ER‐phagy) (Khaminets et al., 2015; Lennemann & Coyne, 2017). Therefore, the role of RHDs in viral infections in animal/human cells may depend on particular RHDs and vary from virus to virus. To address the possible role of RHDs in plant virus infection, a pioneering work was pursued on brome mosaic virus (BMV) using yeast as a surrogate host (Diaz et al., 2010). In yeast mutants whose RHDs are depleted, BMV RNA replication is inhibited by 80%–90%. In BMV‐infected yeast cells, RHPs are recruited for the formation of ER‐derived spherules by interacting with the viral replication factor 1a protein (Diaz & Ahlquist, 2012; Diaz et al., 2010; Diaz & Wang, 2014). Consistently, GmPHD, an RHD protein in soybean (*Glycine max*), has been found to interact with the P3 protein of soybean mosaic virus (SMV) (Cui et al., 2018), and two *Arabidopsis* reticulon‐like B (RTNLB) proteins, AtRTNLB3 and AtRTNLB6, bind to the movement protein (MP) of cauliflower mosaic virus (a DNA virus) and three +ssRNA viruses including potato virus X (PVX), potato mop‐top virus (PMTV) and tobacco mosaic virus (TMV) (Tilsner & Kriechbaumer, 2022). However, the biological relevance of these interactions is yet to be investigated. In a more recent study, Zhang et al. (2023) reported that RTNLB2 binds to the viral protein p23 of beet black scorch virus (BBSV), induces ER membrane curvature, constricts ER tubules to facilitate the assembly of the VRC and promotes BBSV replication in plants (Zhang et al., 2023).

The genus *Potyvirus* within the family *Potyviridae* represents the largest group of known plant‐infecting RNA viruses, including many agriculturally important viruses such as turnip mosaic virus (TuMV) (Yang et al., 2021). The potyviral +ssRNA genome is an RNA of about 10 kb that encodes a large polyprotein and a small polyprotein resulting from a frame shift (Cui & Wang, 2019; Yang et al., 2021). These two polyproteins are cleaved into 11 mature proteins via three viral proteinases (Revers & Garcia, 2015; Yang et al., 2021). Among them, the second 6‐kDa protein, 6K2, is an integral membrane protein that targets and remodels the ER to initiate the formation of ER‐derived membranous vesicles for viral replication (Cotton et al., 2009; Schaad et al., 1997; Wei & Wang, 2008). Like all plant‐infecting viruses, potyviruses employ plasmodesmata (PDs) as a symplasmic route for systemic spread and infection. PDs are a specialized ER‐originated intercellular organelle that enables cytoplasmic and endomembrane continuity between adjacent cells (Lee, 2015). In plants, RTNLs constitute a large gene family, with 21 members (AtRTNLBs) in *Arabidopsis* (Nziengui et al., 2007). Given that RTNLs are ER membrane‐bending proteins and two isoforms in *Arabidopsis*, AtRTNLB3 and AtRTNLB6, are present in the PD proteome for primary PD formation and interact with MPs of several plant viruses (Fernandez‐Calvino et al., 2011; Knox et al., 2015; Kriechbaumer et al., 2015; Tilsner & Kriechbaumer, 2022), we employed TuMV as a model virus to investigate their possible roles in viral infection in plants. Here, we provide evidence that AtRTNLB6 interacts with TuMV CP to promote viral intercellular movement, whereas AtRTNLB3 acts in general as an antiviral factor for TuMV replication via targeting an α‐helix motif in the viral integral membrane protein 6K2.

## 2 RESULTS

### 2.1 AtRTNLB3 and AtRTNLB6 interact with TuMV 6K2 and CP, respectively

**FIGURE 1:**
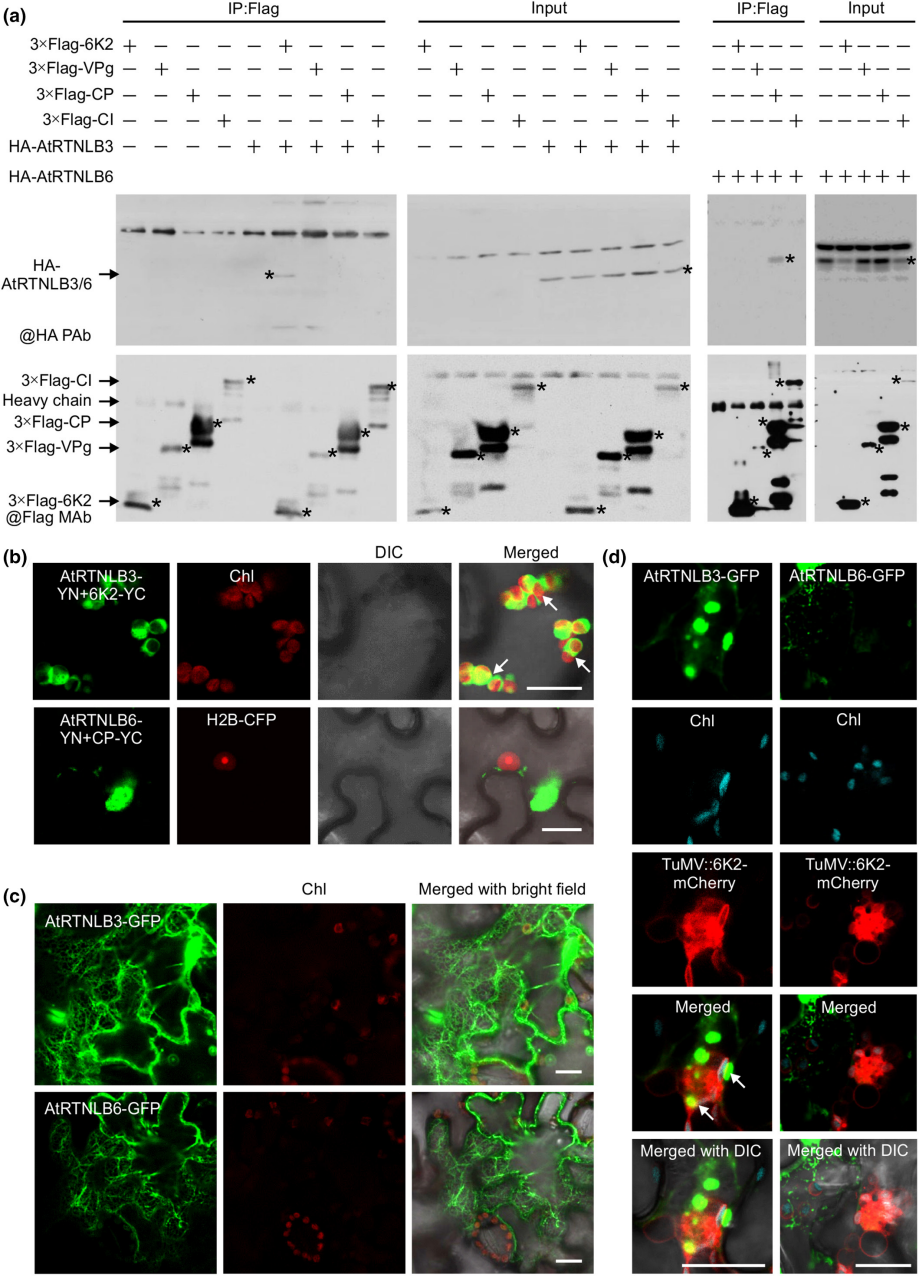
TuMV 6K2 and CP interact with AtRTNLB3 and AtRTNLB6, respectively, and AtRTNLB3 is associated with the 6K2‐induced viral replication complex. (a) Co‐immunoprecipitation experiments to detect in vivo interactions of TuMV proteins 6K2, VPg, CP and CI with AtRTNLB3 or AtRTNLB6 in *Nicotiana benthamiana* cells. Total proteins extracted from the samples were subjected to immunoprecipitation with anti‐FLAG M2 beads, and eluted samples after critical washing were detected by immunoblotting with anti‐HA polyclonal antibodies (@HA PAb) or anti‐FLAG monoclonal antibody (@Flag MAb). Proteins from immunoprecipitated samples are indicated by arrows. The proteins detected corresponding to the predicted size for 3×FLAG‐tagged TuMV proteins (6K2, VPg, CP and CI) and C‐terminal HA‐tagged AtRTNLB3 or AtRTNLB6 are indicated with asterisks. (b) Interactions of AtRTNLB3 or AtRTNLB6 with TuMV proteins in *N. benthamiana* leaf cells examined by the bimolecular fluorescence complementation assay. Colocalizations of the fluorescent signals resulting from the AtRTNLB3 and 6K2 interaction with chloroplasts are indicated with white arrows. H2B‐CFP, a nucleus marker. Scale bars, 20 μm. (c) Confocal microscopy results showing endoplasmic reticulum (ER)‐like structures of AtRTNLB3 and AtRTNLB6 in *N. benthamiana* cells. Scale bars, 20 μm. (d) Subcellular localization of AtRTNLB3‐GFP and AtRTNLB6‐GFP in TuMV‐infected *N. benthamiana* leaf cells. Association of AtRTNLB3 with TuMV 6K2‐induced viral replication complexes is indicated with white arrows. In (b–d), Chl, autofluorescent chloroplasts (red); DIC, differential interference contrast. Scale bars, 20 μm.

To test if any TuMV‐encoded protein(s) interact with AtRTNLB3 or AtRTNLB6, we conducted a bimolecular fluorescence complementation (BiFC) assay in *Nicotiana benthamiana* leaf cells. Among the 11 viral proteins, 6K2, VPg, CP and CI showed positive interaction signals with AtRTNLB3 and AtRTNLB6 (Figure S1). Subsequently, we performed co‐immunoprecipitation (co‐IP) to verify these interactions. 3×FLAG‐tagged TuMV proteins (6K2, VPg, CP and CI) and C‐terminal HA‐tagged AtRTNLB3 or AtRTNLB6 were co‐expressed in *N*. *benthamiana* cells. TuMV 6K2 and CP could be immunoprecipitated with the antibodies against HA (Figure 1a), confirming that AtRTNLB3 specifically interacts with TuMV 6K2 and AtRTNLB6 binds to the CP. Next, we examined the subcellular localization of these two protein–protein interaction complexes in *N*. *benthamiana* leaf cells. We found that the AtRTNLB3 and 6K2 interaction complex, but not the AtRTNLB6 and CP complex, was associated with chloroplasts (Figure 1b). When expressed transiently, AtRTNLB3‐GFP or AtRTNLB6‐GFP showed similar subcellular localizations, both labelling the ER network (mainly ER tubules) in the cytoplasm and forming punctate bodies along the cell wall (Figure 1c, Figure S2a,b). This is consistent with RTNLB's PD and ER localization reported previously (Knox et al., 2015). In addition, AtRTNLB3‐GFP, rather than AtRTNLB6‐GFP, also formed cytoplasmic aggregates associated with autofluorescent chloroplasts in the perinuclear region (Figure S2a). In TuMV‐infected cells, the AtRTNLB3‐labelled structures were associated with viral 6K2‐induced vesicles (Figure 1d), which are a hallmark of potyviral replication sites accommodating VRCs (Cotton et al., 2009; Wei, Huang, et al., 2010; Wei & Wang, 2008).

### 2.2 AtRTNLB3 and AtRTNLB6 play contrasting roles in TuMV infection

The fact that AtRTNLB3 and AtRTNLB6 interact with TuMV viral proteins 6K2 and CP, respectively, renders a possibility that they are involved in TuMV infection. To test this idea, we pursued a genetic approach. We searched the database of the Arabidopsis Biological Resource Center (ABRC, https://abrc.osu.edu/) for possible mutants of *AtRTNLB3* and *AtRTNLB6*. As there were no knockout mutants available, we obtained two knockdown lines SALK_067184 and SALK_118027, designated as *atrtnlb3* and *atrtnlb6* (Huang et al., 2018), respectively. Homozygous mutants were determined by genotyping PCR (Figure S3). Under given growth conditions, *atrtnlb3* showed similar viability and morphology to the wild‐type (WT) *Arabidopsis*, while *atrtnlb6* could not develop fully expanded leaves and displayed a narrow leaf phenotype (Figure S4a). Reverse transcription‐quantitative PCR (RT‐qPCR) analysis confirmed that both *atrtnlb3* and *atrtnlb6* are knockdown mutants (Figure S4b). We also generated a homozygous double mutant *atrtnlb3*/*6* through conventional breeding (Figure S3); the double mutant developed the stunted leaf phenotype, which ws apparently not as severe as *atrtnlb6* (Figure S4a). Furthermore, we generated transgenic *Arabidopsis* plants (Col‐0 ecotype) overexpressing *AtRTNLB3* or *AtRTNLB6* (Figure S5a,b). The resulting transgenic overexpression plants showed no obvious morphological difference with WT plants (Figure S5b).

**FIGURE 2:**
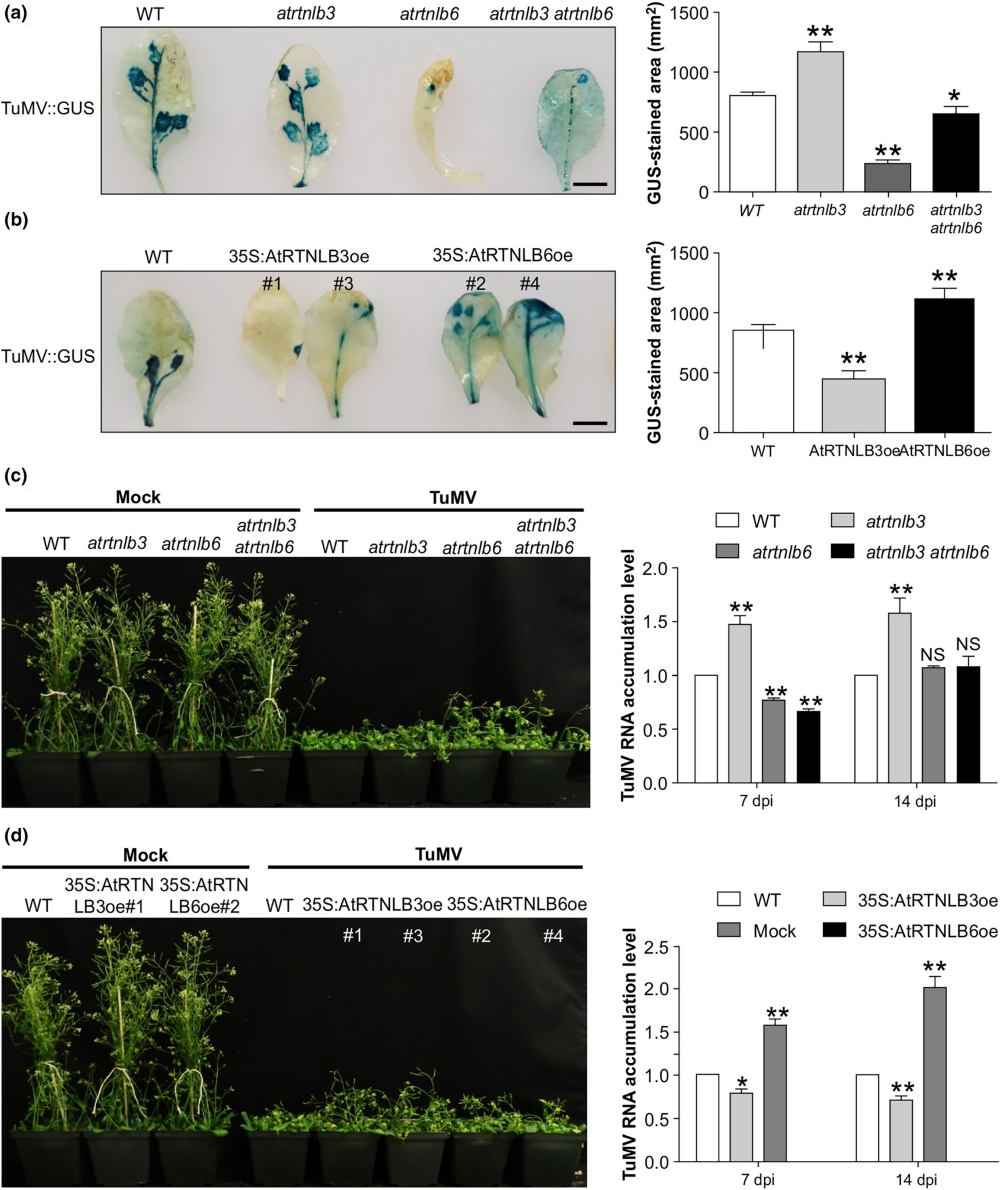
Effects of knockdown and transgenic overexpression of *AtRTNLB3* and *AtRTNLB6* on TuMV infection. (a, b) β‐glucuronidase (GUS) staining of local TuMV‐infected leaves of *Arabidopsis* knockdown mutants (a) and transgenic overexpression plants (b) at 5 days post‐inoculation (dpi). Wild‐type (WT) plants were used as a control. Scale bars, 1.0 cm. GUS‐stained area was quantified using ImageJ software. Data represent means with *SD* of three biological replicates. For each biological replicate of every treatment, 15 inoculated leaves with GUS staining were randomly selected for quantification analysis. This experiment was repeated three times. *p < 0.05, **p < 0.01; NS, not significant. (c, d) TuMV‐infected plants and viral RNA accumulation. TuMV‐induced symptoms were monitored over 4 weeks post‐inoculation. Viral RNA accumulation was quantified by reverse transcription‐quantitative PCR in WT and knockdown mutants at 7 dpi and in WT and transgenic overexpression plants at 14 dpi. The relative TuMV RNA accumulation level is shown. The amount of viral RNA in WT was set to 1. Data represent means with *SD* of three biological replicates. *p < 0.05, **p < 0.01; NS, not significant.

The two single mutants *atrtnlb3* and *atrtnlb6*, the double mutant *atrtnlb3*/*6* and representative transgenic lines overexpressing *AtRTNLB3* (35S:AtRTNLB3oe) or *AtRTNLB6* (35S:AtRTNLB6oe) were challenged with the TuMV recombinant full‐length cDNA infectious clone TuMV::GUS, which was generated by the insertion of a *β‐glucuronidase* (*GUS*) gene between the P1 and HC‐Pro coding sequence of the TuMV full‐length cDNA infectious pCambiaTuMV::GFP (hereafter TuMV::GFP) (Cotton et al., 2009; Huang et al., 2010). We visualized TuMV infection by performing GUS staining. At 5 days post‐infection (dpi), GUS staining was evident on the inoculated leaves of all *Arabidopsis* plants (Figure 2a,b). In comparison with that in WT *Arabidopsis* plants, TuMV infection was remarkably inhibited in the inoculated leaves of *atrtnlb6* knockdown and *AtRTNLB3* overexpression plants (Figure 2a,b). We quantified the GUS‐stained area and found that TuMV infection areas were reduced in *atrtnlb6* single knockdown, *atrtnlb3*/*6* double mutant and *AtRTNLB3* overexpression plants but increased in the *atrtnlb3* mutant or AtRTNLB6 overexpression plants (Figure 2a,b). We monitored the systemic symptom development and the viral accumulation level over 4 weeks post‐inoculation. Compared to WT plants (which developed severe symptoms), *atrtnlb6*, *atrtnlb3/6* and 35S:AtRTNLB3oe plants showed relatively milder symptoms, whereas *atrtnlb3* and 35S:AtRTNLB6oe lines displayed more severe symptoms (Figure 2c,d). Consistently, significant higher levels of viral RNA accumulation were found in *atrtnlb3*, and lower levels of viral RNA accumulation were detected in *atrtnlb6* and *atrtnlb3/6* mutants (Figure 2c). In transgenic plants, overexpression of *AtRTNLB3* inhibited TuMV accumulation, whereas *AtRTNLB6*‐overexpression plants accumulated significant higher levels of TuMV (Figure 2d). Taken together, these results suggest that AtRTNLB3 and AtRTNLB6 play contrasting roles in TuMV infection. AtRTNLB3 inhibits TuMV infection as an antiviral factor, whereas AtRTNLB6 promotes TuMV infection as a proviral factor.

### 2.3 AtRTNLB3 restricts TuMV replication and AtRTNLB6 facilitates viral intercellular movement

**FIGURE 3:**
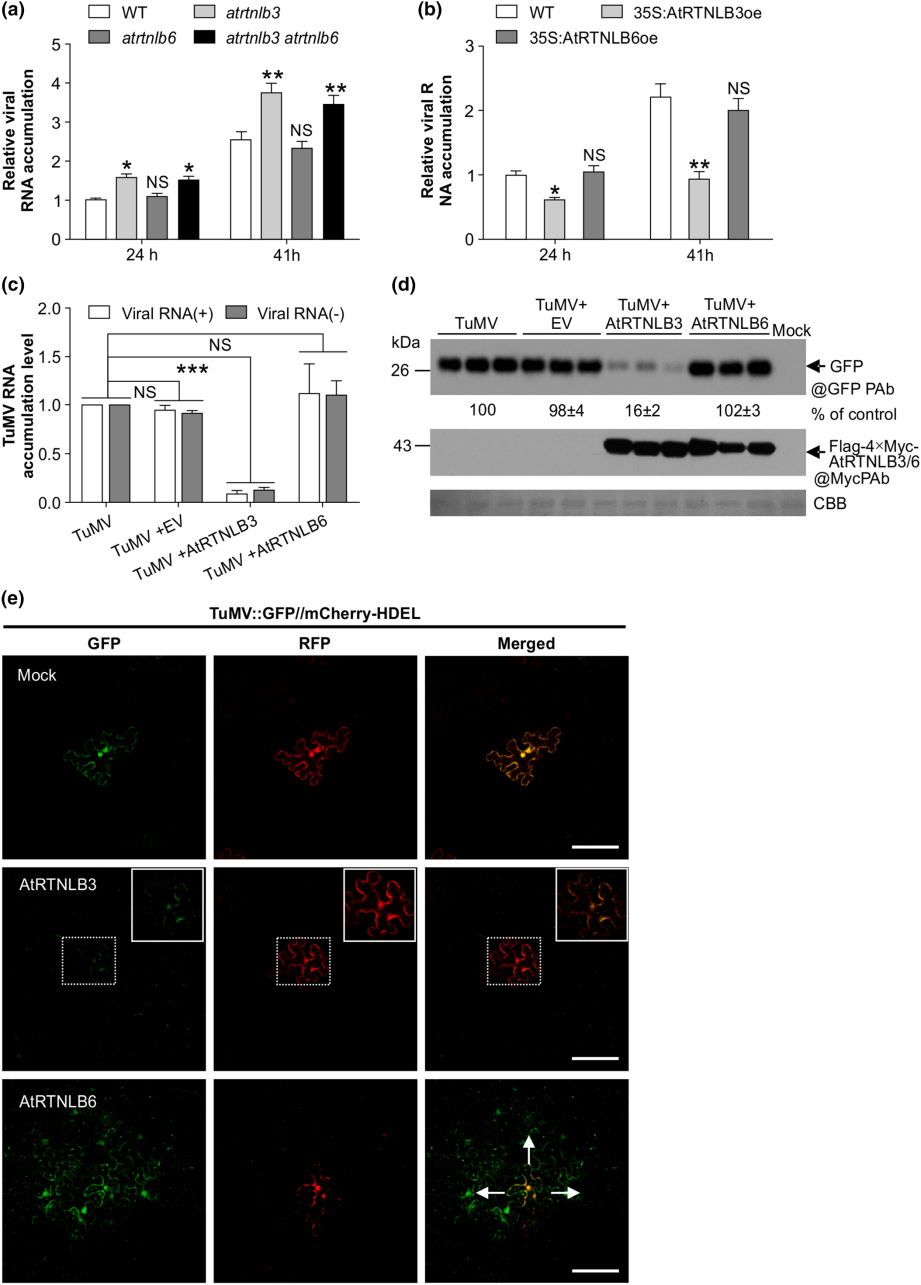
AtRTNLB3 and AtRTNLB6 participate in TuMV replication and intercellular movement, respectively. (a, b) TuMV infection assay in protoplasts from wild‐type plants (WT), *AtRTNLB3* or *AtRTNLB6* knockdown mutants (a) and transgenic overexpression plants (b). Data represent means with *SD* of three biological replicates. *p < 0.05, **p < 0.01; NS, not significant. (c) Effects of transient overexpression of *AtRTNLB3* or *AtRTNLB6* on TuMV infection in *Nicotiana benthamiana* leaf tissues. Reverse transcription‐quantitative PCR assay of positive‐sense viral RNA ([+]RNA) and negative‐sense viral RNA ([−]RNA) accumulation in local leaves of *N*. *benthamiana* plants agroinfiltrated with the TuMV infectious full‐length cDNA clone TuMV::GFP (TuMV), TuMV + empty vector (EV) and TuMV + AtRTNLB3 or AtRTNLB6 at 2 days post‐agroinfiltration (dpai). *F‐box* transcript was used as an internal control. Data represent means with *SD* of three biological replicates. ***p < 0.001; NS, not significant. (d) Immunoblotting analyses of GFP accumulation derived from the infectious clone TuMV::GFP in the local infiltrated leaves of *N*. *benthamiana* plants co‐expressing *AtRTNLB3* or *AtRTNLB6* at 3 dpai. The relative amount of the GFP protein is indicated. CBB, Coomassie Brilliant Blue R‐250‐stained RuBisCO large subunit serves as a loading control. (e) Confocal microscopy analysis of viral intercellular movement in *N. benthamiana*. pCB301TuMV‐GFP//mCherry‐HDEL was agroinfiltrated into with *N. benthamiana* leaf tissues with mock buffer (upper panels), AtRTNLB3 (middle panels) and AtRTNLB6 expression vector (lower panels). Images were taken at 3 dpai. Inset is an enlarged view of the dashed area. Cells labelled by double fluorescence indicate primarily infected ones and those highlighted by GFP alone represent secondarily infected cells. Arrows point to the direction of viral movement. Scale bars, 100 μm.

To explore the molecular mechanisms underlying the roles of AtRTNLB3 and AtRTNLB6 in TuMV infection, we conducted a transfection assay in protoplasts essentially as described previously (Deng et al., 2015; Wu et al., 2018) to observe if viral replication is affected by knockdown or overexpression of *AtRTNLB3* and *AtRTNLB6*. Protoplasts from *Arabidopsis* knockdown mutants and overexpression plants were transfected with the TuMV infectious clone TuMV::GFP, and the viral RNA accumulation level was monitored by RT‐qPCR at 24 and 41 hours post‐transfection (hpt). At both time points, the TuMV accumulation level was significant higher in *atrtnlb3* or *atrtnlb3/6* mutants but significantly lower in the *AtRTNLB3* overexpression line, compared to the control (Figure 3a,b). Knockdown or overexpression of *AtRTNLB6* did not affect viral accumulation in *Arabidopsis* protoplasts. These results suggest that AtRTNLB3 suppresses TuMV replication and AtRTNLB6 apparently does not affect viral replication.

To confirm the effects of overexpression of *AtRTNLB3* or *AtRTNLB6* on viral infection in another plant species, *AtRTBLB3* or *AtRTBLB6* was transiently expressed via agroinfiltration in *N*. *benthamiana* leaf tissues concomitantly inoculated with the TuMV infectious clone TuMV::GFP. The accumulation level of viral genomic RNA (positive sense, +) and negative‐sense (−) viral RNA was monitored by RT‐qPCR in inoculated leaves at 2 days post‐agroinfiltration (dpai). At this time point, all viral RNAs were produced in primarily infected cells as our previous studies have shown that potyviral intercellular spread occurs after 3–4 dpai (Cui et al., 2017; Park et al., 2017; Wu et al., 2018, 2020). Consistent with results of *Arabidopsis* transgenic overexpression plants, transient co‐expression of *AtRTNLB6* in *N. benthamiana* did not affect viral RNA accumulation but transient co‐expression of *AtRTNLB3* did inhibit viral RNA accumulation (Figure 3c). Immunoblotting analysis of accumulation levels of GFP resulting from the recombinant TuMV infectious clone also confirmed that transient expression of *AtRTNLB3* in *N. benthamiana* leaf cells restricted viral infection expressing AtRTNLB3, but transient expression of *AtRTNLB6* did not (Figure 3d).

Because AtRTNLB3 and AtRTNLB6 are also localized to PD (Knox et al., 2015), it is possible that they are involved in TuMV intercellular movement. To test this possibility, we inoculated *N. benthamiana* leaves transiently expressing *AtRTNLB3* or *AtRTNLB6* with the vector PCB301TuMV‐GFP//mCherry‐HDEL (OD_600_ of agrobacterial culture, 0.001). This viral vector could be used to distinguish primarily and secondarily infected cells as it contains two expression cassettes: one for the generation of a recombinant infectious TuMV tagged by GFP and the other for the expression of mCherry‐HDEL (Dai et al., 2020). Primarily infected cells would emit two colours (GFP and mCherry) of fluorescence, whereas secondarily infected cells resulting from viral intercellular spread would radiate GFP fluorescence only. As mentioned above, potyviral intercellular movement is usually evident after 3–4 dpai. Indeed, at 3 dpai, the recombinant virus was only present in primarily infected cells in the mock‐treated leaf tissues (Figure 3e). No viral cell‐to‐cell movement occurred in leaf tissues co‐expressing *AtRTNLB3* either (Figure 3e). However, secondarily infected cells (emitting GFP fluorescence only) were observed when co‐expressed with *AtRTNLB6* (Figure 3e). These results suggest that AtRTNLB6 facilitates TuMV intercellular movement.

### 2.4 AtRTNLB3 suppresses 6K2 self‐interaction and replication vesicle formation by targeting the α‐helix motif ^42^LRKSM^46^ in 6K2

**FIGURE 4:**
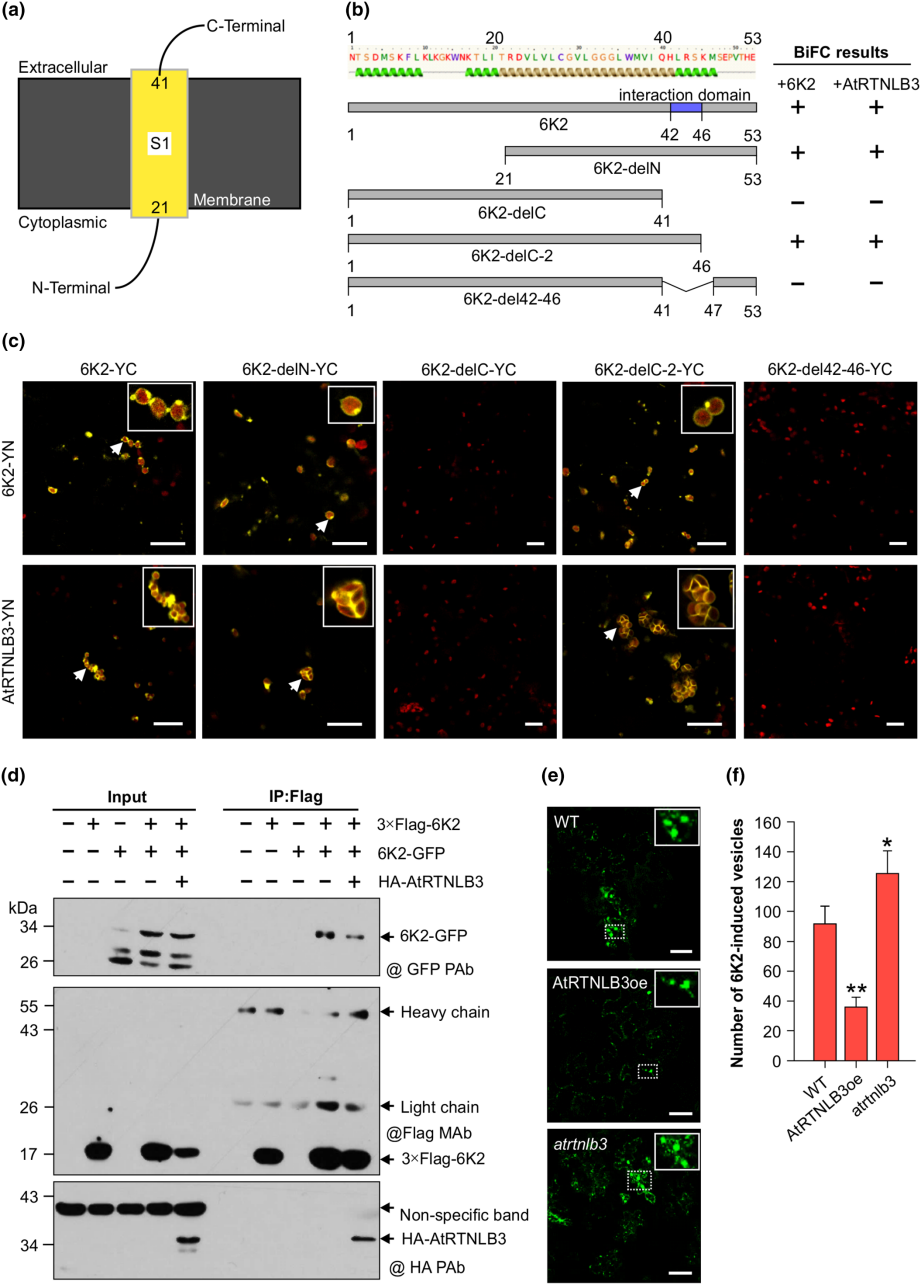
The α‐helix motif in TuMV 6K2 is responsible for interactions with AtRTNLB3 and 6K2 self‐interactions and is essential for viral replication. (a) Predicated transmembrane structure (S1, yellow) in TuMV 6K2 Phyre 2.0 server. Numbers in the yellow boxes indicate the start and end positions of the transmembrane helix in TuMV 6K2, respectively. (b) Schematic representation of the full‐length and truncated TuMV 6K2 used for mapping of interaction regions (left) and summary of bimolecular fluorescence complementation (BiFC) results of 6K2 or AtRTNLB3 with 6K2 and its four deletion mutants. 6K2‐delN, amino acids (aa) 21–53; 6K2‐delC, aa 1–41; 6K2‐delC‐2, aa 1–46. +, positive reaction; −, negative reaction. (c) BiFC assay of the interactions of TuMV 6K2 or AtRTNLB3 with 6K2 and its four deletion mutants in *Nicotiana benthamiana* leaf cells. Typical colocalizations of vesicles with the outer membrane of chloroplasts are indicated with white arrows. Inset is a magnified image of the chloroplast‐associated aggregates. Chl, autofluorescent chloroplasts. Scale bars, 20 μm. (d) TuMV 6K2 and AtRTNLB3 compete to bind TuMV 6K2. Co‐immunoprecipitation experiments were conducted to examine the possible competition between TuMV 6K2 and AtRTNLB3 for binding to 6K2. Total proteins from the harvested samples were subjected to immunoprecipitation with anti‐FLAG M2 beads, and eluted samples after critical washing were detected by immunoblotting using anti‐GFP or anti‐HA polyclonal antibodies (@GFP PAb and @HA PAb) and anti‐FLAG monoclonal antibody (@Flag MAb). Bands corresponding to the target proteins are indicated by black arrows. (e, f) Effects of knockdown and overexpression of *AtRTNLB3* on 6K2‐induced vesicle formation. TuMV::6K2‐GFP infectious clone was agroinfiltrated into wild‐type Col‐0, *atrtnlb3* and AtRTNLB3oe *Arabidopsis* plants. Representative images of 6K2‐induced vesicles in different plants are shown in (e). Inset is a magnified view of the dashed area. Numbers of 6K2‐tagged vesicles were counted from 30 frames per treatment at 7 days post‐agroinfiltration (f). Scale bars, 40 μm. Data represent means with *SD* of three biological replicates. *p < 0.05, **p < 0.01.

The observation that AtRTNLB3 negatively regulates TuMV replication is different from the functional role of RHDs in BMV infection in yeast (Diaz et al., 2010) and that of RTNLB2 in BBSV infection in plants (Zhang et al., 2023). AtRTNLB3 interacts with 6K2, which is a self‐interacting, integral membrane protein that induces ER proliferations for the formation of membranous replication vesicles for potyviral genome replication (Cotton et al., 2009; Restrepo‐Hartwig & Carrington, 1994; Schaad et al., 1997; Wei, Huang, et al., 2010; Wei & Wang, 2008). Thus, we investigated whether the AtRTNLB3 and 6K2 interaction interferes with 6K2 function. Based on structure modelling, TuMV 6K2 has a central transmembrane domain (amino acids [aa] 21–41), an N‐terminal tail (located in the cytosol) and a C‐terminal tail in the ER lumen or vesicle (Figure 4a), and contains α‐helix regions beside transmembrane regions (Figure 4b). We conducted BiFC and co‐IP assays and confirmed the self‐interaction of TuMV 6K2 (Figure 4c,d). To map the region in 6K2 that is required for the self‐interaction and the 6K2 and AtRTNLB3 interaction, we generated two 6K2 truncated mutants, named 6K2‐delN (aa 21–53) and 6K2‐delC (aa 1–41), by deletion of the N‐terminal aa 1–20 and C‐terminal aa 42–53 (Figure 4b). In the BiFC assay, neither 6K2 nor AtRTNLB3 interacted with 6K2‐delC, but both of them bound to 6K2‐delN, suggesting that the very C‐terminal fragment (aa 42–53) is required for the 6K2 self‐interaction as well as its interaction with AtRTNLB3 (Figure 4b,c). Because this fragment contains an α‐helix motif (^42^LRSKM^46^) (Figure 4b), we determined if this motif plays any role in these interactions. Two more truncated mutants deleting aa 47–53 (named 6K2‐delC‐2) and the α‐helix motif ^42^LRSKM^46^ (6K2del42‐46) were constructed. BiFC assay showed that 6K2‐delC‐2 retained the ability to bind to 6K2 and AtRTNLB3 but 6K2del42‐46 lost the ability to interact with them (Figure 4b,c), suggesting that the α‐helix motif ^42^LRSKM^46^ is indispensable for the 6K2 self‐interaction as well as the 6K2–AtRTNLB3 interaction. Given that the α‐helix motif of 6K2 is required for both interactions, we questioned whether co‐expression of AtRTNLB3 affects the 6K2 self‐interaction. We conducted co‐IP using protein extracts from *N. benthamiana* leaf tissues co‐expressing TuMV 6K2‐GFP and 3 × FLAG‐6K2 with or without HA‐AtRTNLB3. We found that 3 × FLAG‐6K2 indeed captured lower amounts of 6K2‐GFP when co‐expressing with HA‐AtRTNLB3 (Figure 4d), suggesting AtRTNLB3 interferes with the 6K2 self‐interaction, probably through competition for the binding motif. To assess if the AtRTNLB3–6K2 interaction also plays a negative role on the formation of 6K2‐induced vesicles, we examined the number of 6K2‐labelled vesicles in WT, *atrtnlb3* and AtRTNLB3oe plants infected with a recombinant TuMV, pCambiaTuMV::6K2‐GFP (Cotton et al., 2009; Huang et al., 2010). This recombinant virus contains an extra copy of 6K2 tagged by GFP so that the 6K2‐containing VRCs can be visualized by confocal microscopy. We found that in *AtRTNLB3* overexpression leaf cells, the number of 6K2 vesicles was significantly lower, compared with that in WT plants at 7 dpai (Figure 4e,f). Conversely, the number of 6K2 vesicles was significantly increased in *atrtnlb3* plants. Taken together, these results suggest that overexpression of *AtRTNLB3* suppresses 6K2 self‐interaction and vesicle induction, probably by targeting the α‐helix motif ^42^LRSKM^46^ in 6K2.

### 2.5 Overexpression of *AtRTNLB3* probably promotes the selective degradation of the ER and ER chaperone protein calnexin

**FIGURE 5:**
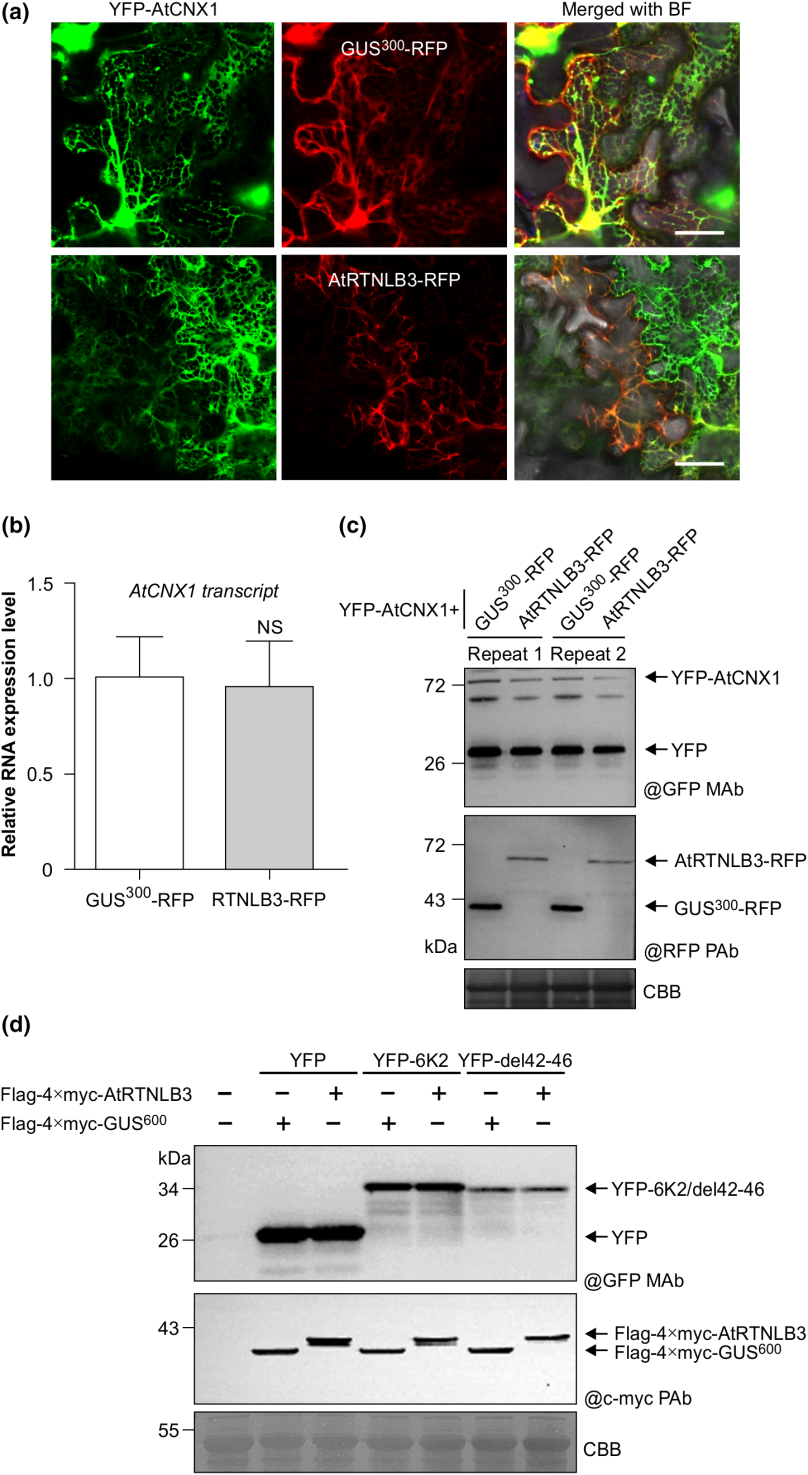
Overexpression of *AtRTNLB3* induces the degradation of the endoplasmic reticulum (ER) and ER chaperone protein calnexin. (a) Confocal microscopy visualization of YFP‐AtCNX1 co‐expressed with AtRTNLB3‐RFP or control GUS^300^‐RFP in *Nicotiana benthamiana* cells. Pictures were taken at 2 days post‐agroinfiltration (dpai). Scale bars, 20 μm. (b) Reverse transcription‐quantitative PCR assay of *AtCNX1* RNA level from samples in (a). *F‐box* transcript was used as an internal control. Data represent means with *SD* of five biological replicates. NS, not significant. (c) Western blot analysis of YFP‐AtCNX1 accumulation under different treatments from samples in (a). Total proteins were extracted and subjected to immunoblotting analysis using anti‐GFP monoclonal or anti‐RFP polyclonal antibodies (@GFP MAb, @RFP PAb) as the primary antibody. CBB, Coomassie Brilliant Blue R‐250‐stained RuBisCO large subunit serves as a loading control. (d) Immunoblotting detection of free YFP, YFP‐6K2 and YFP‐6K2‐del42‐46, when co‐expressed with FLAG‐4×myc‐AtRTNLB3 or the control FLAG‐4×myc‐GUS^600^ in *N*. *benthamiana* cells. GUS^600^, a 600 bp fragment of the cDNA sequence for β‐glucuronidase (GUS). Samples were harvested from infiltrated regions at 2 dpai. Total proteins were extracted and subjected to immunoblotting analysis using anti‐GFP monoclonal or anti‐c‐myc polyclonal antibodies (@GFP MAb, @c‐myc PAb) as the primary antibody. CBB, Coomassie Brilliant Blue R‐250‐stained RuBisCO large subunit serves as a loading control.

A recent study in mammalian cells has shown that overexpression of *RTN3* triggers fragmentation of ER tubules and subsequently ER‐phagy (Grumati et al., 2017). The authors also provided evidence that RTN3 actually is an ER‐phagy receptor for the selective degradation of ER tubules (Grumati et al., 2017). This prompted us to explore whether transient overexpression of *AtRTNLB3* also affects ER stability. To this end, AtCNX1, a known ER chaperone protein calnexin, was included in this study. Confocal microscopy results showed that both the ER structure and YFP‐AtCNX1 fluorescence were largely diminished in *N. benthamiana* leaf cells overexpressing AtRTNLB3‐RFP, compared to the control GUS^300^‐RFP (here a truncated GUS was fused to the N terminus of RFP) (Figure 5a). RT‐qPCR detected similar RNA expression levels of *AtCNX1* in *N. benthamiana* leaf tissues overexpressing AtRTNLB3‐RFP and GUS^300^‐RFP (Figure 5b). However, immunoblotting analysis revealed a reduced accumulation of YFP‐AtCNX1 under the treatment of RTNLB3‐RFP compared with the control GUS^300^‐RFP (Figure 5c). These data suggest that overexpression of AtRTNLB3 may induce the degradation of the ER and ER‐associated protein calnexin. To examine whether AtRTNLB3 can directly target 6K2 for degradation, we conducted a transient expression assay by co‐expression of YFP‐6K2 and FLAG‐4×myc‐RTNLB3 in *N. benthamiana* leaves. A truncated GUS fusion protein FLAG‐4×myc‐GUS^600^ was used as a control. Immunoblotting detected similar levels of YFP‐6K2 in leaf tissues expressing FLAG‐4×myc‐AtRTNLB3 or the control FLAG‐4×myc‐GUS^600^ (Figure 5d). Moreover, co‐expression with FLAG‐4×myc‐AtRTNLB3 also did not apparently reduce the protein level of the 6K2 mutant YFP‐6K2‐del42‐46 (Figure 5d). These results suggest that overexpression of AtRTNLB3 induces the degradation of the ER and ER chaperone protein calnexin but does not affect 6K2 accumulation.

### 2.6 Three amino acids L42, R43 and K45, in the α‐helix motif are essential for the 6K2 and AtRTNLB3 interaction

**FIGURE 6:**
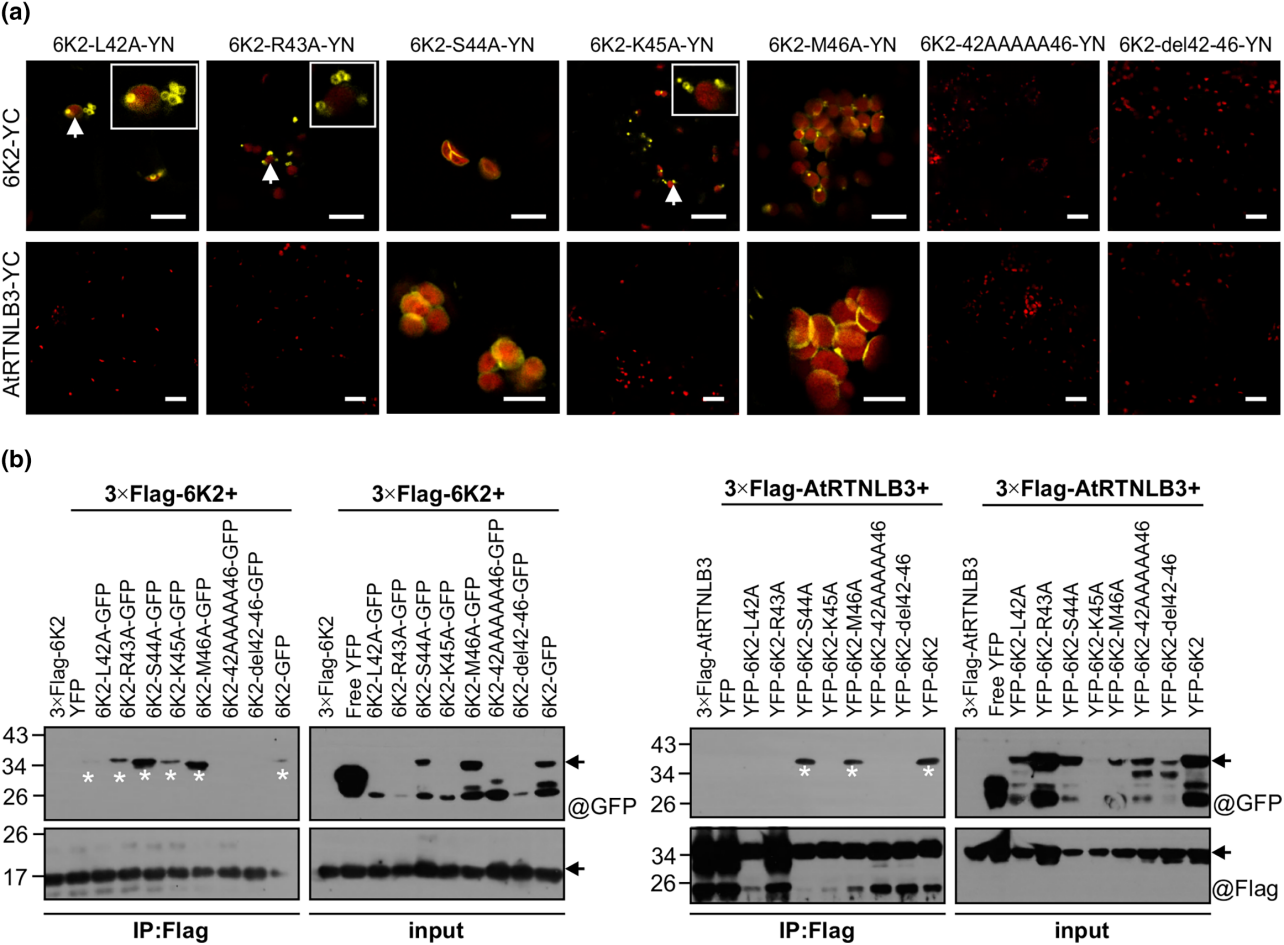
Effects of substitutions of the α‐helix motif in TuMV 6K2 with alanine on the interactions of 6K2 with AtRTNLB3. (a) Bimolecular fluorescence complementation analysis of the interactions of TuMV 6K2 mutants with wild‐type 6K2 or AtRTNLB3. Abnormal vesicles resulting from the interactions between 6K2 mutants and wild‐type 6K2 are indicated with white arrows, and inset is a magnified image. Scale bars, 20 μm. (b) Co‐immunoprecipitation analysis of the interactions of TuMV 6K2 mutants with wild‐type 6K2 or AtRTNLB3 in *Nicotiana benthamiana* cells. Total proteins from the harvested samples were subjected to immunoprecipitation with anti‐FLAG M2 beads (Sigma), and eluted samples after critical washing were detected by immunoblotting using anti‐GFP polyclonal antibodies (@GFP) or anti‐FLAG monoclonal antibody (@Flag). Bands corresponding to the target proteins are indicated by arrows. The proteins of interest from eluted products were indicated by white asterisks.

To identify the aa residues in the α‐helix motif critical for the interaction with AtRTNLB3, we made multiple alignments of aa sequences of 6K2 from different potyviruses. We found that this α‐helix motif is quite divergent and only the aa at the position 42 is relatively conserved (Figure S6). We then conducted alanine (A)‐scanning mutagenesis by substitution of each of the five amino acids in this motif, and the resulting mutants were designated as 6K2‐L42A, 6K2‐R43A, 6K2‐S44A, 6K2‐K45A and 6K2‐M46A. Additionally, a mutant named 6K2‐42AAAAA46, in which all the five amino acids were mutated to A, was also generated. Then, we carried out a BIFC assay to test if these mutants interact with 6K2 and AtRTNLB3. As shown (Figure 6a), all single aa substitution mutants could interact with 6K2, but three of them, namely 6K2‐L42A, 6K2‐R43A and 6K2‐K45A, lost the ability to interact with AtRTNLB3. Mutants 6K2del42‐46 and 6K2‐42AAAAA46 could not bind to either AtRTNLB3 or wild‐type 6K2. The BiFC protein–protein interaction results were further confirmed by co‐immunoprecipitation analyses (Figure 6b). Based on these data, we conclude that three residues in the α‐helix motif, that is, L42, R43 and K45, are essential for the 6K2 and AtRTNLB3 interaction.

### 2.7 L42, R43 and K45 in 6K2 are critical for TuMV replication and infectivity

**FIGURE 7:**
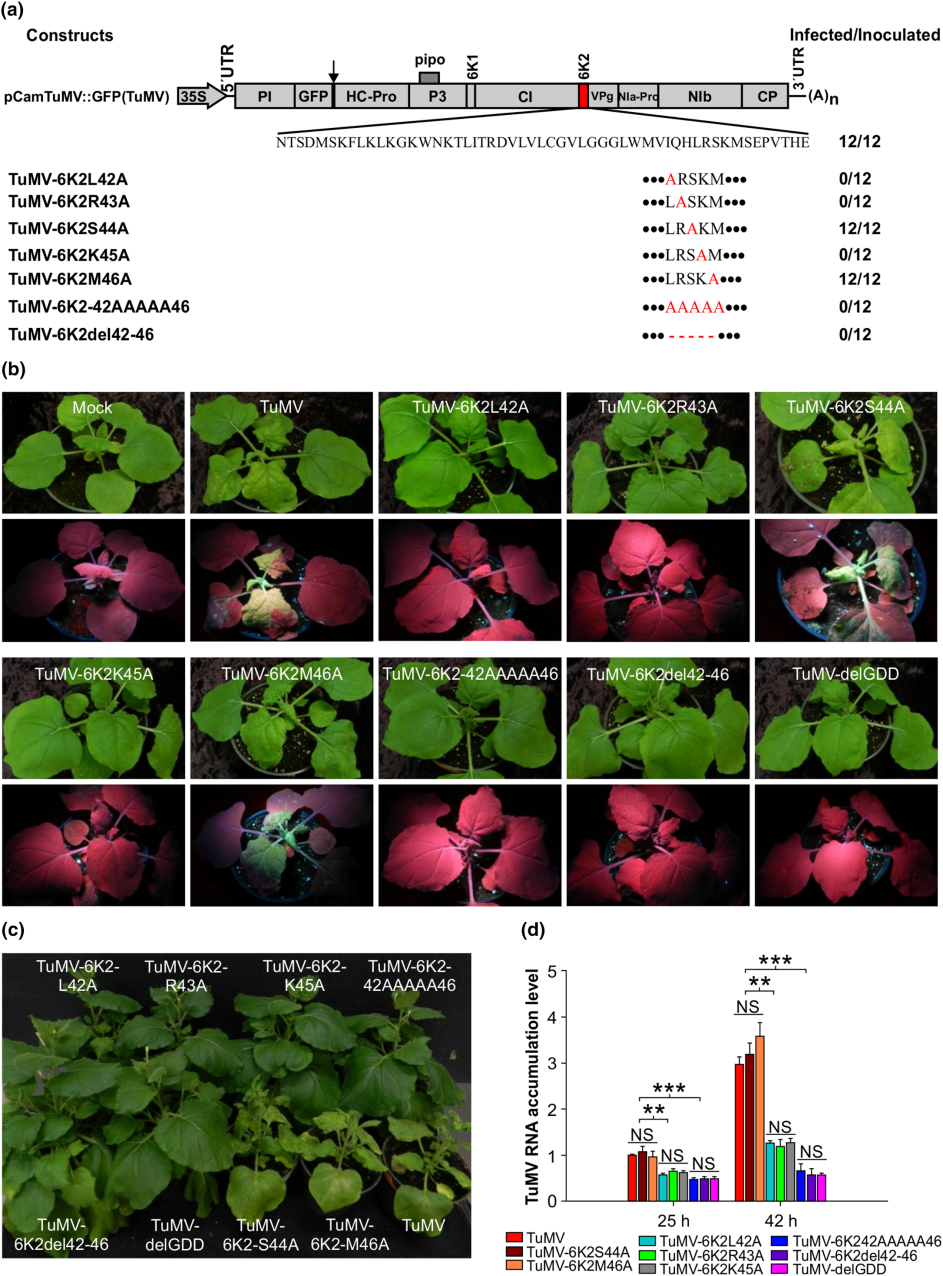
The effect of alanine substitution or deletion in the alpha‐helix motif of 6K2 on TuMV infectivity. (a) Diagram of the TuMV infectious clone and its mutants in the 6K2 region. Black arrow indicates the artificial added protease sites between GFP and HC‐Pro. Black dots at the beginning or end of the α‐helix motif in 6K2 represent the original flank sequences. The horizontal dashed line represents deletion of amino acids. For each independent infection experiment, 12 plants were inoculated with the TuMV wild‐type infectious clone (TuMV) or each of the 6K2 mutants. Number of infected plants is given. (b) Phenotypes of *Nicotiana benthamiana* plants agroinfiltrated with TuMV wild‐type infectious clone (TuMV) and its 6K2 mutants. Underneath is the image with GFP fluorescence under UV light. Photographs were taken at 7 days post‐agroinfiltration (dpai). (c) Phenotypes of *N*. *benthamiana* plants agroinfiltrated with TuMV wild‐type infectious clone and its 6K2 mutants at 23 dpai. (d) Reverse transcription‐quantitative PCR detection of the viral RNA accumulation level in *Arabidopsis* protoplasts transfected with TuMV wild‐type infectious clone and different 6K2 mutants. *F‐box* was used as an internal control. Data represent means with *SD* of three biological replicates. **p < 0.01, ***p < 0.001; NS, not significant.

To determine if the α‐helix motif ^42^LRKSM^46^ and aa in this motif are essential for TuMV infection, we mutated this motif in the TuMV infectious clone TuMV::GFP and constructed seven TuMV mutants, designated as TuMV‐6K2L42A, TuMV‐6K2‐R43A, TuMV‐6K2‐S44A, TuMV‐6K2‐K45A, TuMV‐6K2‐M46A, TuMV‐6K2‐42AAAAA46 and TuMV‐6K2del42‐46 (Figure 7a). A TuMV replication‐deficient mutant in which the conserved GDD motif in NIb is deleted (Hong & Hunt, 1996), designated as TuMV‐delGDD, was used as a control. Wild‐type TuMV and all mutants were agroinfiltrated into *N. benthamiana* leaves at an OD_600_ of 0.1. At 7 dpai, only plants inoculated with TuMV‐6K2‐S44A, TuMV‐6K2‐M46A and wild‐type TuMV showed typical TuMV‐infected symptoms and systemic infection, as judged by visual observation and fluorescent signals in systemic leaves under UV light (Figure 7a,b). At 23 dpai, inoculation with the other five mutants did not induce symptoms, and no systemic infection was evident (0/12) (Figure 7c). These results were confirmed by RT‐qPCR to quantify viral RNA accumulation levels at 28 dpai (Figure S7).

To further confirm the role of this motif in TuMV replication, we conducted protoplast transfection assay. Protoplasts were isolated from WT *Arabidopsis* and then transfected with WT TuMV::GFP, TuMV mutants and TuMV‐delGDD. Viral genomic RNA was quantified by RT‐qPCR. We found that at both time points examined, the viral RNA level of three mutants TuMV‐6K2L42A, TuMV‐6K2‐R43A and TuMV‐6K2‐K45A was significantly lower than that of TuMV::GFP but much higher than TuMV‐delGDD (Figure 7d), suggesting that mutation of any of these three residues significantly reduced viral replication. In contrast, mutation of the residue S44 or M46 did not affect viral replication. TuMV‐6K2‐42AAAAA46 and TuMV‐6K2del42‐46 lost the ability to replicate as only very low levels of viral RNA were detected, comparable to that of TuMV‐delGDD (resulting from the activity of a 35S promoter). Consistently, when expressed alone, both the 6K2‐42AAAAA46 and 6K2del42‐46 mutants lost 6K2's ability to target chloroplasts in *N. benthamiana* leaf cells (Figure S8). Taken together, these results indicate that the α‐helix motif ^42^LRSKM^46^ and specifically its three residues (L42, R43 and K45) are critical for TuMV replication and viability.

## 3 DISCUSSION

In mammals, the ER membrane‐bending proteins RTNs have been shown to function as a proviral factor for several viruses (Aktepe et al., 2017; Diaz et al., 2010; Tang et al., 2007) and in some other cases, they may play an antiviral role (Khaminets et al., 2015; Lennemann & Coyne, 2017; Wu et al., 2014). The role of RTNLs in plant virus infection remains poorly understood. In this study, we investigated the possible involvement of AtRTNLB3 and AtRTNLB6 in TuMV infection. These two isoforms have been found to be present in the PD proteome and are recruited to the cell plate during the formation of primary PD in *Arabidopsis* (Fernandez‐Calvino et al., 2011; Knox et al., 2015; Kriechbaumer et al., 2015). We set out from screening for possible interactions between viral proteins and these two RTNLBs. Our BiFC experiments identified four TuMV proteins 6K2, VPg, CP and CI that interacted with AtRTNLB3 and AtRTNLB6 (Figure S1). Our co‐IP assay only confirmed two interactions (AtRTNLB3 and 6K2; AtRTNLB6 and CP) in all three independent experiments. It is possible that other interactions identified by BiFC might be false‐positive or too weak to be detected by co‐IP. Because AtRTNLB3 and AtRTNLB6 were confirmed to interact with 6K2 and CP, respectively, we conducted TuMV infectivity assays with *Arabidopsis atrtnlb3* and *atrtnlb6* mutants as well as with transgenic *AtRTNLB3* and *AtRTNLB6* overexpression lines. We found that AtRTNLB3 negatively regulated TuMV infection, whereas AtRTNLB6 promoted TuMV infection (Figure 2). We further performed experiments to explore possible underlying mechanisms. We concluded that AtRTNLB6 functions as a proviral factor by promoting TuMV intercellular movement, whereas AtRTNLB3 restricts TuMV infection by suppressing viral replication (Figure 3).

Potyviral CP is not required for viral replication but indispensable for viral intercellular movement (Dai et al., 2020). In addition to CP, other potyviral proteins (namely, P3N‐PIPO and CI) also have essential MP function (Wang, 2021). P3N‐PIPO is a PD‐located protein that targets CI to PD to form special conical structures for potyviral passage (Wei, Zhang, et al., 2010), highly likely in the form of virions (Dai et al., 2020; Wang, 2021). It is possible that the AtRTNLB6 and CP interaction is involved in the intracellular translocation of virions to PD. It is also possible that AtRTNLB6 is recruited by CP to modify the central desmotubule for potyviral passage through PD. This assumption is supported by the previous finding that while both AtRTNLB3 and AtRTNLB6 are associated with primary PD in the mature cell wall and colocalize with the MP of TMV at PD, only AtRTNLB6 is associated with the central desmotubule (Knox et al., 2015). Recently, AtRTNLB6 and AtRTNLB3 have been shown to interact with MPs of several plant viruses including a DNA virus and three +ssRNA viruses (Tilsner & Kriechbaumer, 2022). It would be interesting to determine whether either AtRTNLB3 or AtRTNLB6 or both are involved in cell‐to‐cell movement of these viruses or not.

As mentioned above, the finding that AtRTNLB3 restricts TuMV infection was unexpected. Therefore, we conducted experiments to explore possible underlying mechanisms. Because AtRTNLB3 interacted with 6K2 (Figure 1), which is the only potyviral protein having the ability to induce membranous vesicles essential for potyviral replication (Cotton et al., 2009; Wei, Huang, et al., 2010; Wei & Wang, 2008), we characterized the effect of this interaction on the formation of 6K2‐induced vesicles. 6K2 is known as a single‐pass integral membrane protein with the cytosol‐located N‐terminal tail, a central TMD and a C‐tail that protrudes into the ER lumen or vesicles (Jiang et al., 2015). In this study, we found that 6K2 self‐interacted via the α‐helix motif in the C‐tail and deletion of the α‐helix motif in TuMV 6K2 abolished both 6K2 self‐interaction and its ability to induce the formation of membranous vesicles (Figure 4). AtRTNLB3 also interacted with this motif (Figure 6). Overexpression of AtRTNLB3 compromised 6K2 self‐interaction and VRC formation (Figure 4) and TuMV replication (Figure 3). This is consistent with the antiviral role of RTN3 in flavivirus infection (Wu et al., 2014). In this case, RTN3 interacts with the non‐structural protein of HCV and negatively regulates viral replication by inhibiting NS4B self‐interaction (Wu et al., 2014).

Being the largest membranous organelle, the ER functions as the major membrane source for the formation of autophagosomes as being remodelled as replication sites by many +ssRNA viruses. Under stress, the ER initiates autophagy and further induces selective autophagy, which regulates ER turnover (ER‐phagy) (Bernales et al., 2007). The negative role of ER‐phagy mediated by RHDs in animal virus replication has been revealed in recent years (Grumati et al., 2017; Lennemann & Coyne, 2017). In this study, we report that AtRTNLB3 overexpression induced the degradation of the ER and the ER chaperone protein calnexin (Figure 5). Notably, 6K2 was not degraded in the cells overexpressing AtRTNLB3, suggesting 6K2 may have the ability to escape from AtRTNLB3‐mediated protein degradation. This result further supports the hypothesis that RTNLB3 impairs 6K2 self‐interaction to suppress TuMV infection, rather than affecting the stability of the 6K2 protein. 6K2 itself can activate the unfolded protein response (UPR) (Zhang et al., 2015) and selective autophagy in a UPR‐dependent manner to promote viral infection (Li et al., 2020). The precise ER‐phagy signalling pathway in plants is unclear (Grumati et al., 2018). Further study is needed to understand if the 6K2–AtRTNLB3 interaction contributes to the suppression of the antiviral role of AtRTNLB3 and how AtRTNLB3 overexpression activates ER‐phagy and further regulates viral infection.

**FIGURE 8:**
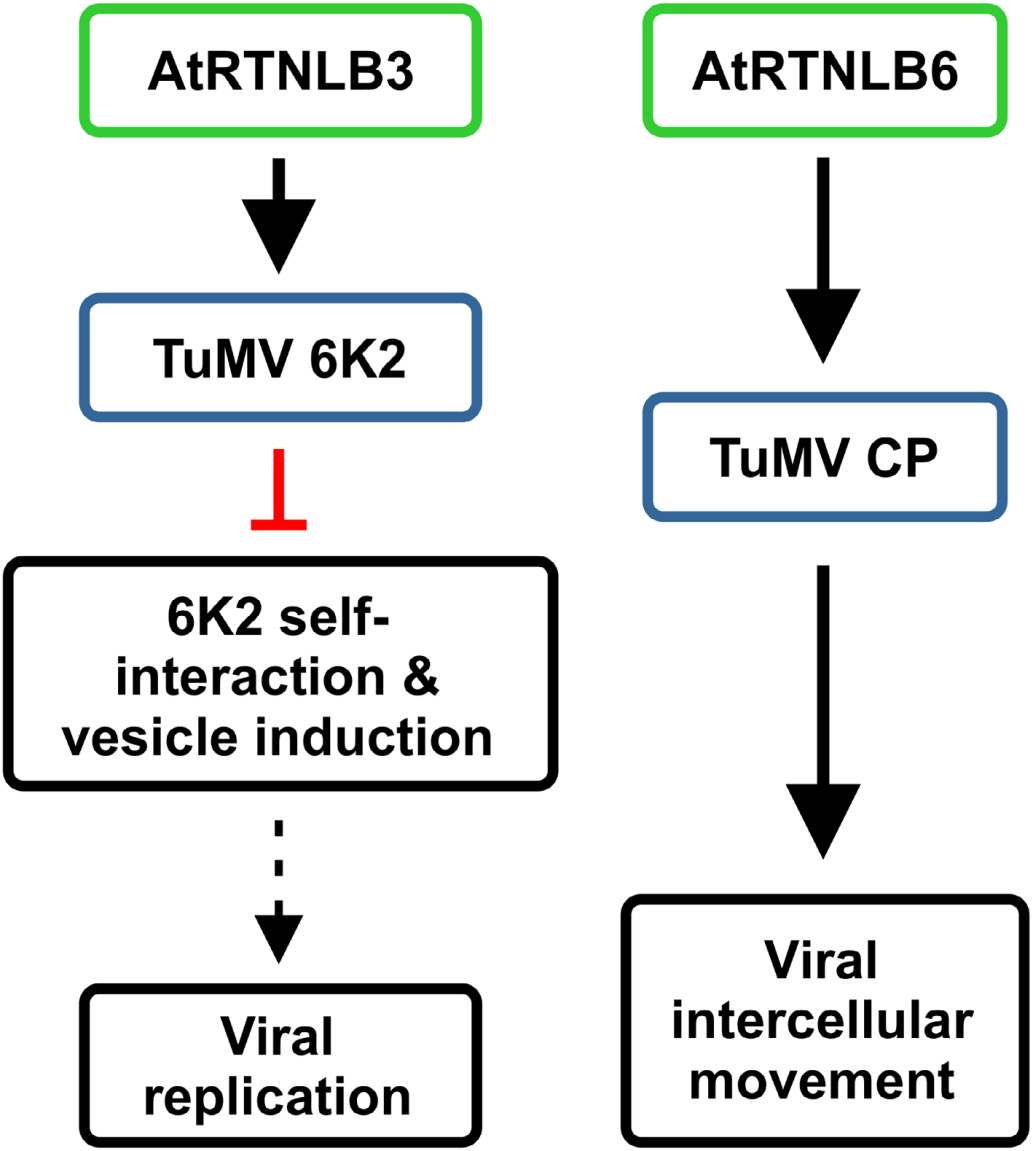
A proposed model of RTNLB3 and RTNLB6 in TuMV infection. After the translation of the TuMV viral genome, the viral integral membrane protein 6K2 self‐interacts at the endoplasmic reticulum (ER) and induces vesicle formation. The ER‐localized AtRTNLB3 binds to TuMV 6K2 at the α‐helix motif of 6K2 to interfere with the 6K2 self‐interaction and further suppresses vesicle formation and viral genome replication. In contrast, the ER‐localized protein AtRTNLB6 interacts with TuMV CP to enhance viral intercellular movement.

Based on the above discussion, we propose a model for the possible involvement of AtRTNLB3 and AtRTNLB6 in TuMV infection (Figure 8). After translation of the viral genome, 6K2 induces ER membrane proliferations to form 6K2‐induced vesicles that house the VRC. This process requires 6K2 self‐interactions via the α‐helix motif located in the ER lumen. AtRTNLB3 binds to 6K2 at the α‐helix motif, interferes with 6K2 self‐interactions and further inhibits vesicle formation to restrict viral infection. On the other hand, AtRTNLB6 interacts with the CP and targets virions to PD to promote vial intercellular movement.

## 4 EXPERIMENTAL PROCEDURES

### 4.1 Plant materials and growth conditions

The *Arabidopsis* mutants *atrtnlb3* (SALK_067184) and *atrtnlb6* (SALK_118027) were obtained from the Arabidopsis Biological Resource Centre (Ohio State University). Homozygous lines were screened by PCR genotyping using the primer pairs listed in Table S1. The *atrtnlb3 atrtnlb6* double mutant was generated via genetic crossing. Unless stated otherwise, all *Arabidopsis* plants were grown in a growth chamber with a 14 h photoperiod and a relative humidity of 75% at 23/21°C (light/dark). *N. benthamiana* plants were grown in a growth room with a 16 h photoperiod and a relative humidity of 75% at 22°C.

### 4.2 Generation of transgenic plants

The coding sequence of *AtRTNLB3* or *AtRTNLB6* was cloned into the plant binary vector pBA‐FLAG‐4×Myc‐DC (Zhu et al., 2011) using Gateway cloning technology (Thermo Fisher Scientific). The resulting vector was introduced into the WT *Arabidopsis* plant (ecotype Columbia‐0) by the floral‐dip method (Zhang et al., 2006). Transformants were screened by direct spraying of solutions containing 20 mg/L glufosinate‐ammonium and confirmed by PCR.

### 4.3 Gene cloning, plasmid construction and expression

Phusion high‐fidelity DNA polymerase (New England Biolabs) was used for PCR with primer sets listed in Table S1. All vectors were verified by DNA sequencing. Coding sequences of *AtRTNLB3* and *AtRTNLB6* from *A. thaliana* Columbia‐0 were amplified from *Arabidopsis* cDNA. The homologous genes of these two *Arabidopsis* genes in *N. benthamiana* were retrieved from its draft genome (Bombarely et al., 2012) and amplified from its cDNA. Coding sequences of TuMV genes were amplified from the infectious clone TuMV::GFP (GenBank accession number: EF028235.1). The amplified fragments were inserted into the entry vector pDONR221 (Thermo Fisher Scientific), followed by recombination into plant expression vectors via LR reactions. *AtRTNLB3* and *AtRTNLB6* were recombined into pEarleyGate103 giving C‐terminal GFP and into pEarleyGate201 to generate N‐terminal HA‐fusion proteins (Earley et al., 2006). For BiFC assay, the TuMV and host genes were transferred into the modified pEarleyGate201‐nYFP or pEarleyGate202‐cYFP vector (Lu et al., 2010). To construct the TuMV:GUS infectious clone, a β‐glucuronidase (GUS) reporter gene was cloned from pBI121 vector and inserted between P1 and HC‐Pro to replace the *GFP* gene in TuMV::GFP infectious clone. All plant expression vectors were electroporated into *Agrobacterium tumefaciens* GV3101. The GV3101 cells harbouring the expression constructs were resuspended with the infiltration buffer and infiltrated into *N. benthamiana* leaves as previously described (Wei et al., 2013).

### 4.4 Reverse transcription‐quantitative PCR

Total RNA was isolated with an RNeasy Plant Mini Kit (Qiagen) and treated with DNase I (Invitrogen). After removal of DNase I, first‐strand cDNA synthesis was done using Superscript III reverse transcriptase (Invitrogen) and an oligo(dT) primer (Invitrogen) as previously described (Cheng et al., 2017). For quantification of viral positive‐sense and negative‐sense RNA, the protocol was followed as previously described (Cui & Wang, 2016). *F‐box* was used as an internal standard (Lilly et al., 2011). Primers were listed in Table S1.

### 4.5 Site‐directed mutagenesis

Site‐directed mutagenesis in the 6K2 gene was performed with specific primer pairs listed in Table S1 and using Phusion high‐fidelity DNA polymerase. For the construction of mutated TuMV infectious clones, the wild‐type TuMV infectious clone pCambiaTuMV::GFP was used as the parental virus as previously described with some modifications (Deng et al., 2015). Briefly, a 6K2‐containing fragment (about 5300 bp) between two unique restriction endonuclease sites (SnaBI and MluI) in pCambiaTuMV::GFP clone was used as a template. The introduction of alanine (A) into the specific positions in 6K2 was conducted with specific primer pairs using a QuickChange site‐directed mutagenesis kit (Stratagene). The fragment generated by overlapping extension PCR was then digested with the restriction enzymes (SnaBI and MluI), gel purified and ligated into the corresponding sites of pCambiaTuMV::GFP clone. All mutated 6K2 genes and TuMV infectious clones were confirmed by DNA sequencing.

### 4.6 BiFC and confocal microscopy

BiFC assay was conducted as described previously (Wu et al., 2018, 2020). Different combinations of vectors containing target genes for interaction identification and combinations of vectors as negative controls were agroinfiltrated into *N. benthamiana* leaves. The agroinfiltrated tissue was imaged at 48–72 h post‐inoculation by using a TCS SP2 inverted confocal microscope (Leica Microsystems), equipped with a 63× water‐corrected objective in multitrack mode as previously described (Li et al., 2018). The sequential scanning mode was applied for co‐imaging of different fluorescent proteins. All BiFC experiments including negative controls were repeated at least three times.

### 4.7 Co‐immunoprecipitation assays

Co‐immunoprecipitation experiments were performed as previously described with some modifications (Win et al., 2011). The examined genes were fused with 3×FLAG or GFP tag and expressed in *N. benthamiana* leaves. Immunoprecipitation was done 2.5 days post‐infiltration by using anti‐FLAG M2 affinity gel (Sigma). Samples were loaded onto an SDS‐PAGE gel followed by western blotting analysis using anti‐FLAG and anti‐GFP IgG (Sigma). Blots were incubated with horseradish peroxidase‐conjugated secondary antibody (Sigma), and signals were detected by enhanced chemiluminescence (ECL, Millipore). All co‐IPs were performed at least three times.

### 4.8 Protoplast isolation and transfection

Protoplasts were isolated from healthy *N*. *benthamiana* or *Arabidopsis* plants (Yoo et al., 2007) and transfected with plasmids as previously described (Wu et al., 2018).

### 4.9 GUS staining

For GUS staining, local infected or mock‐treated leaves were collected, submerged in X‐Gluc solution, vacuum‐infiltrated and then incubated at 37°C overnight (15 h). Stained tissues were decolourized (for green tissues clear of chlorophyll) in 70% ethanol for 4 h and then photographed.

### 4.10 Data analysis

Statistical graphs were generated using GraphPad Prism (v. 5.0) software. Multiple sequence alignments were conducted on the T‐coffee web server and viewed by using Jalview software (Waterhouse et al., 2009). The schematic diagrams for protein domains and viral genome were drawn by using the IBS tool (Liu et al., 2015). ImageJ was used to quantify the GUS‐stained area, average integrated density values of bands on immunoblots and 6K2‐tagged vesicles as the manual instruction.

Sequence data from this article can be found in the Arabidopsis Genome database under accession numbers EF028235.1 for TuMV‐GFP, AT1G64090 for AtRTNLB3, AT3G61560 for AtRTNLB6 and AT5G61790 for AtCNX1.

## CONFLICT OF INTEREST STATEMENT

The authors declare no conflict of interest.

## Supporting information


**FIGURE S1.** Bimolecular fluorescence complementation assay of the interactions of AtRTNLB3 or AtRTNLB6 with four TuMV proteins (6K2, VPg, CP and CI) in *Nicotiana benthamiana* leaf cells. Two pairs NbRTN2‐YN + 6K2‐YC TuMV and NbRTN3‐YN + CP‐YC were used as negative controls. Different combinations of expression vectors were agroinfiltrated into *N. benthamiana* leaf tissues. The infiltrated area was visualized under a confocal microscope, and the image was taken at 48 h post‐winfiltration. In TuMC CP‐YC + AtRTNLB3‐YN or AtRTNLB6‐YN and four bottom images, scale bars = 20 μm. In other images, scale bars = 10 μm.

**FIGURE S2.** Subcellular localization of AtRTNLB3‐GFP or AtRTNLB6‐GFP in *Nicotiana benthamiana* cells. (a) Colocalization assay of AtRTNLB3‐GFP or AtRTNLB6‐GFP with autofluorescent chloroplasts. Association of cellular aggregates formed by AtRTNLB3 with autofluorescent chloroplasts (Chl) is indicated with white arrowhead. Inset is an enlarged view of the aggregates indicated by the arrowhead. Scale bars, 20 μm. (b) Colocalization assay of AtRTNLB3‐GFP or AtRTNLB6‐GFP with the endoplasmic reticulum (ER) marker mCherry‐HDEL. Expression vector AtRTNLB3‐GFP or AtRTNLB6‐GFP was co‐agroinfiltrated with an mCherry‐HDEL expression vector into *N. benthamiana* leaf tissues. The infiltrated area was visualized under a confocal microscope, and the image was taken at 48 h post‐infiltration. Typical ER structures are co‐highlighted by AtRTNLB3‐GFP and mCherry‐HDEL or AtRTNLB6‐GFP and mCherry‐HDEL. Scale bar = 20 μm.

**FIGURE S3.** PCR genotyping of homozygous mutants *atrtnlb3*, *atrtnlb6* and *atrtnlb3 atrtnlb6*. WT, Col‐0; NC, negative control.

**FIGURE S4.** Characterization of T‐DNA mutants. (a) Phenotype of the *Arabidopsis* ecotype Col‐0 (WT) and homozygous T‐DNA mutants *atrtnlb3*, *atrtnlb6* and *atrtnlb3 atrtnlb6* at 12 days under normal growth condition. Scale bars, 2 cm. (b) Reverse transcription‐quantitative PCR assay on the mRNA expression level of *AtRTNLB3* and *AtRTNLB6* in the corresponding mutants.

**FIGURE S5.** Molecular characterization of *AtRTNLB3* and *AtRTNLB6* overexpression plants. (a) Verification of overexpression of *AtRTNLB3* or *AtRTNLB6* in different transgenic *Arabidopsis* lines by western blot. CBB, Coomassie Brilliant Blue‐stained gel used as a loading control. (b) Phenotype of 14‐day‐old *Arabidopsis* wild‐type Col‐0 (WT) and the transgenic overexpression plants under normal growth condition.

**FIGURE S6.** Multiple‐sequence alignments of the 6K2 protein sequences of 16 potyvirus members. The 6K2 sequences of different potyviruses were retrieved from the NCBI GenBank database under the GenBank accession numbers: *Turnip mosaic virus* (TuMV; EF028235.1), *Sweet potato feathery mottle virus* (SPFMV; AB465608.1), *Tobacco vein banding mosaic virus* (TVBMV; EU734432.1), *Plum pox virus* (PPV; NC_001445.1), *Lily mottle virus* (LMoV; AB570195.1), *Bean yellow mosaic virus* (BYMV; JX173278.1), *Potato virus A* (PVA; AJ131402.1), *Papaya ringspot virus* (PRSV; X97251.1), *Chilli veinal mottle virus* (ChiVWV; AJ972878.1), *Tobacco etch virus* (TEV; NC_001555.1), *Potato virus Y* (PVY; AJ439544.2), *Watermelon mosaic virus* (WMV; EU660581.1), *Soybean mosaic virus* (SMV; AF241739.1), *Bean common mosaic virus* (BCMV; HQ229995.1), *Zucchini mosaic virus* (ZYMV; AF014811.2) and *Cowpea aphid‐borne mosaic virus* (CABMV; NC004013.1). The alpha‐helix motif in 6K2 is indicated with red rectangle.

**FIGURE S7.** Reverse transcription‐quantitative PCR detection of the viral CP RNA accumulation level in the upper new leaves of *Nicotiana benthamiana* agroinfiltrated with TuMV and different mutants at 28 days post‐agroinfiltration. *F‐box* was used as an internal control. Data represent means with *SD* of three biological replicates. NS, no significant difference.

**FIGURE S8.** Subcellular localization of 6K2‐GFP and its mutants 6K2‐del42‐46‐GFP or 6K2‐42AAAAA46‐GFP in *Nicotiana benthamiana* leaves. Chloroplast autofluorescence is shown in red. Scale bar = 20 μm.

**TABLE S1.** List of primers used in this study.

## Data Availability

All data generated and analysed during this study are included in this published article and its Supporting Information files.
