# Supplementary material for: Two plant membrane‐shaping reticulon‐like proteins play contrasting complex roles in turnip mosaic virus infection
Source: Mol Plant Pathol. 2024 Oct 16;25(10):e70017. doi: 10.1111/mpp.70017 (PMC11481689; doi:10.1111/mpp.70017)
Supplement: Supplementary file 1 — FIGURE S1. Bimolecular fluorescence complementation assay of the interactions of AtRTNLB3 or AtRTNLB6 with four TuMV proteins (6K2, VPg, CP and CI) in Nicotiana benthamiana leaf cells. Two pairs NbRTN2‐YN + 6K2‐YC TuMV and NbRTN3‐YN + CP‐YC were used as negative controls. Different combinations of expression vectors were agroinfiltrated into N. benthamiana leaf tissues. The infiltrated area was visualized under a confocal microscope, and the image was taken at 48 h post‐winfiltration. In TuMC CP‐YC + AtRTNLB3‐YN or AtRTNLB6‐YN and four bottom images, scale bars = 20 μm. In other images, scale bars = 10 μm. [file MPP-25-e70017-s002.docx]

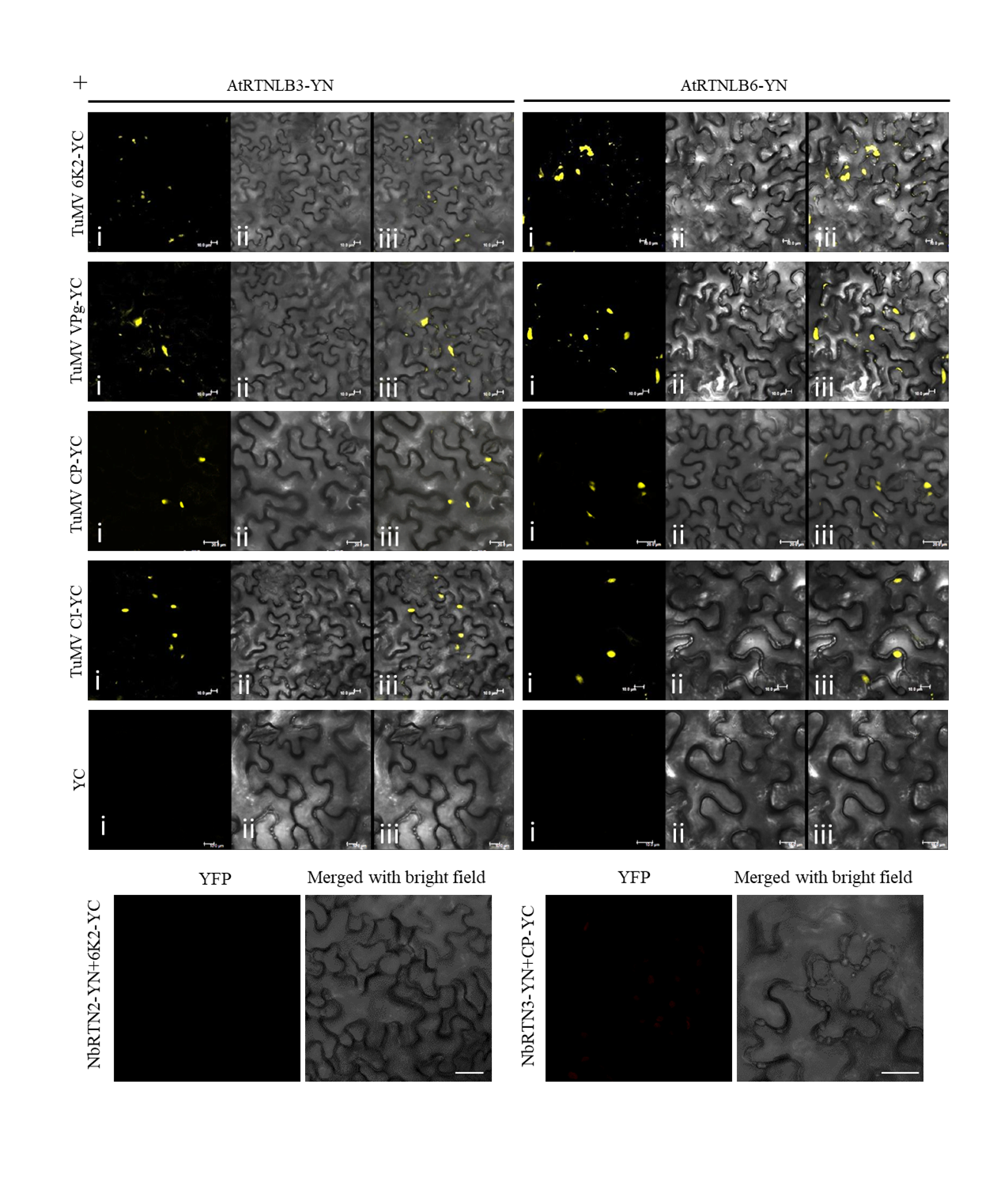


**Figure S1.** Interactions between AtRTNLB3 or AtRTNLB6 and four TuMV proteins (6K2, VPg, CP, and CI) in *Nicotiana benthamiana* leaf cells examined by BiFC. Two pairs NbRTN2-YN + 6K2-YC TuMV and NbRTN3-YN + CP-YC were used as negative controls. Different combinations of expression vectors were agroinfiltrated into *N. benthamiana* leaf tissues. The infiltrated area was visualized under a confocal microscope and image was taken at 48 hpi. In TuMC CP-YC+ AtRTNLB3-YN or AtRTNLB6-YN and four bottom images, scale bars = 20 um. In other images, scale bars = 10 um.
