# Supplementary material for: Two plant membrane‐shaping reticulon‐like proteins play contrasting complex roles in turnip mosaic virus infection
Source: Mol Plant Pathol. 2024 Oct 16;25(10):e70017. doi: 10.1111/mpp.70017 (PMC11481689; doi:10.1111/mpp.70017)
Supplement: Supplementary file 2 — FIGURE S2. Subcellular localization of AtRTNLB3‐GFP or AtRTNLB6‐GFP in Nicotiana benthamiana cells. (a) Colocalization assay of AtRTNLB3‐GFP or AtRTNLB6‐GFP with autofluorescent chloroplasts. Association of cellular aggregates formed by AtRTNLB3 with autofluorescent chloroplasts (Chl) is indicated with white arrowhead. Inset is an enlarged view of the aggregates indicated by the arrowhead. Scale bars, 20 μm. (b) Colocalization assay of AtRTNLB3‐GFP or AtRTNLB6‐GFP with the endoplasmic reticulum (ER) marker mCherry‐HDEL. Expression vector AtRTNLB3‐GFP or AtRTNLB6‐GFP was co‐agroinfiltrated with an mCherry‐HDEL expression vector into N. benthamiana leaf tissues. The infiltrated area was visualized under a confocal microscope, and the image was taken at 48 h post‐infiltration. Typical ER structures are co‐highlighted by AtRTNLB3‐GFP and mCherry‐HDEL or AtRTNLB6‐GFP and mCherry‐HDEL. Scale bar = 20 μm. [file MPP-25-e70017-s007.docx]

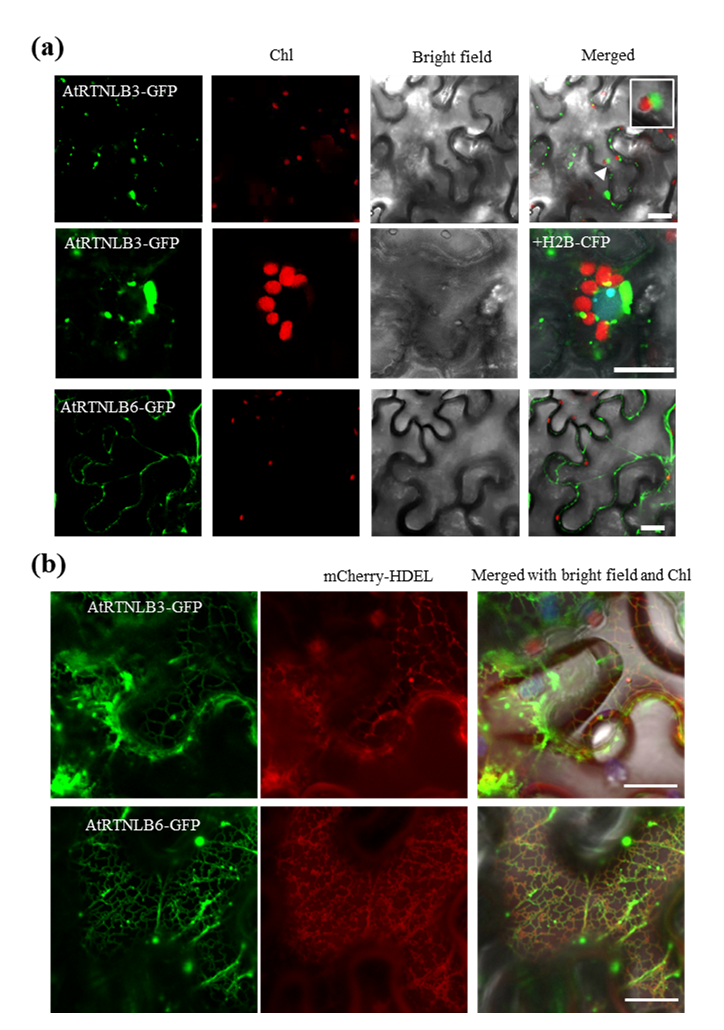


**Figure S2.** Subcellular localization of AtRTNLB3-GFP or AtRTNLB6-GFP in *N*. *benthamiana* cells. (a) Colocalization assay of AtRTNLB3-GFP or AtRTNLB6-GFP with auto-fluorescent chloroplasts. Association of cellular aggregates formed by AtRTNLB3 with auto-fluorescent chloroplasts (Chl) is indicated with white arrow head. Inset is an enlarged view of the aggregates indicated by the arrow head. Scale bars, 20 µm. (b) Colocalization assay of AtRTNLB3-GFP or AtRTNLB6-GFP with the ER marker mCherry-HDEL. Expression vector AtRTNLB3-GFP or AtRTNLB6-GFP was co-agroinfiltrated with an mCherry-HDEL expression vector into *N. benthamiana* leaf tissues. The infiltrated area was visualized under a confocal microscope and image was taken at 48 hpi. Typical ER structures are co-highlighted by AtRTNLB3-GFP and mCherry-HDEL or AtRTNLB6-GFP and mCherry-HDEL. Scale bar = 20 um.
