# Supplementary material for: Two plant membrane‐shaping reticulon‐like proteins play contrasting complex roles in turnip mosaic virus infection
Source: Mol Plant Pathol. 2024 Oct 16;25(10):e70017. doi: 10.1111/mpp.70017 (PMC11481689; doi:10.1111/mpp.70017)
Supplement: Supplementary file 3 — FIGURE S3. PCR genotyping of homozygous mutants atrtnlb3, atrtnlb6 and atrtnlb3 atrtnlb6. WT, Col‐0; NC, negative control. [file MPP-25-e70017-s004.docx]

*atrtnlb3*

*atrtnlb6*

LP+RP

*atrtnlb3 T-DNA insertion detection*

*atrtnlb6 T-DNA insertion detection*


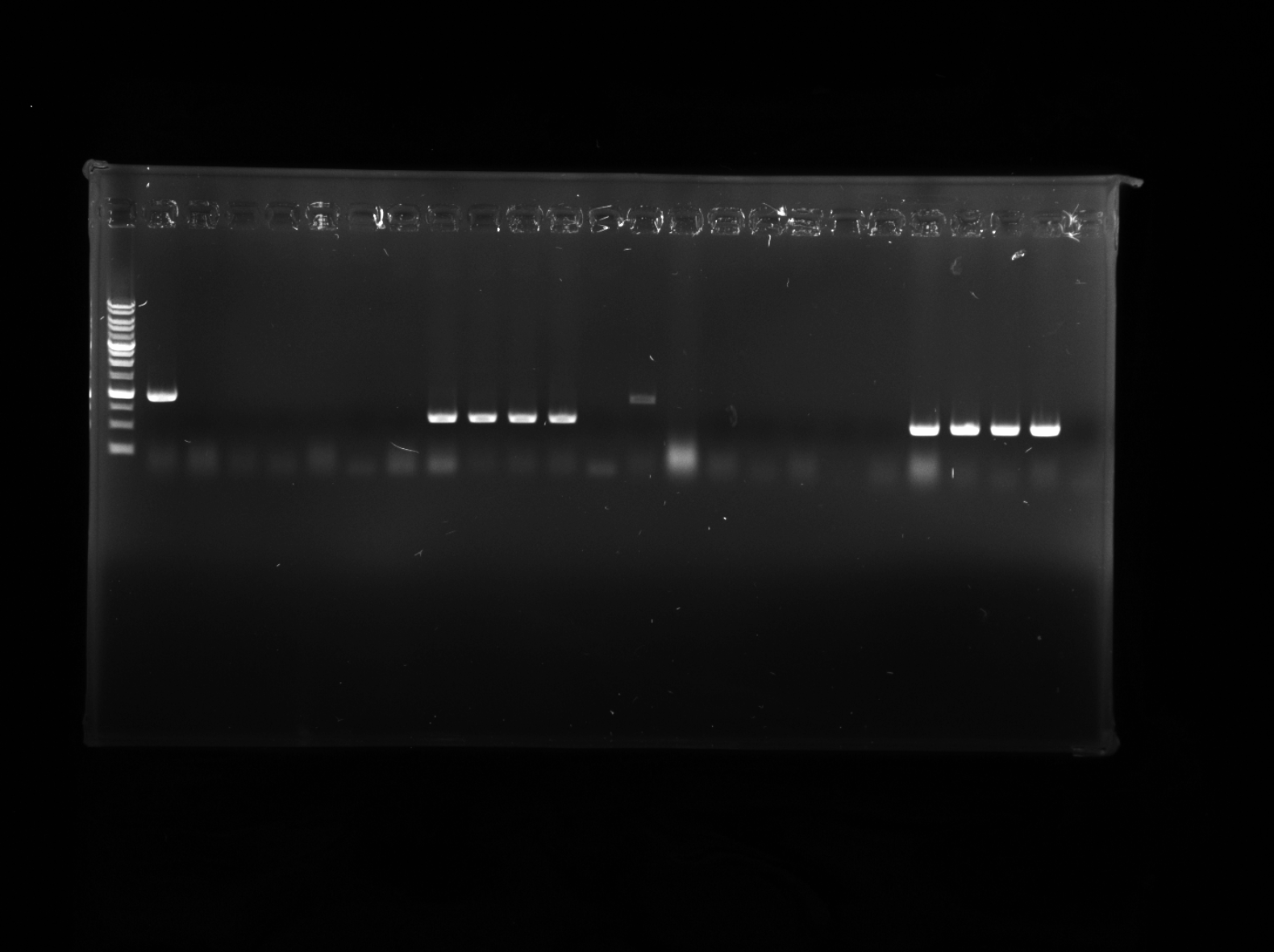


WT

NC

WT

NC

WT

NC

WT

NC

*atrtnlb3*

*atrtnlb3*

*atrtnlb3*

*atrtnlb6*

*atrtnlb3*

*atrtnlb6*

*atrtnlb3*

*atrtnlb6*

*atrtnlb6*

*atrtnlb6*

LP+RP

LBb1.3+RP

LBb1.3+RP

1000 bp

750 bp

500 bp

**Figure S3.** PCR genotyping of homozygous mutants *atrtnlb3*, *atrtnlb6*, and *atrtnlb3* *atrtnlb6*. WT, Col-0; NC, negative control.
