# Supplementary material for: Two plant membrane‐shaping reticulon‐like proteins play contrasting complex roles in turnip mosaic virus infection
Source: Mol Plant Pathol. 2024 Oct 16;25(10):e70017. doi: 10.1111/mpp.70017 (PMC11481689; doi:10.1111/mpp.70017)
Supplement: Supplementary file 4 — FIGURE S4. Characterization of T‐DNA mutants. (a) Phenotype of the Arabidopsis ecotype Col‐0 (WT) and homozygous T‐DNA mutants atrtnlb3, atrtnlb6 and atrtnlb3 atrtnlb6 at 12 days under normal growth condition. Scale bars, 2 cm. (b) Reverse transcription‐quantitative PCR assay on the mRNA expression level of AtRTNLB3 and AtRTNLB6 in the corresponding mutants. [file MPP-25-e70017-s008.docx]

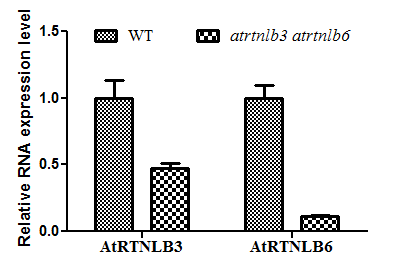

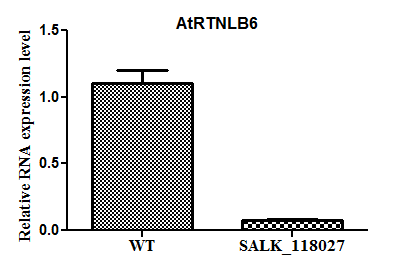

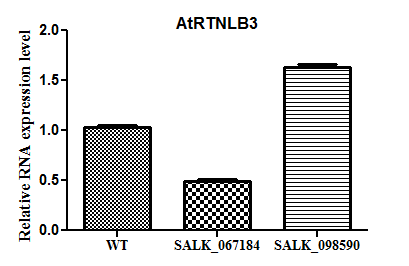

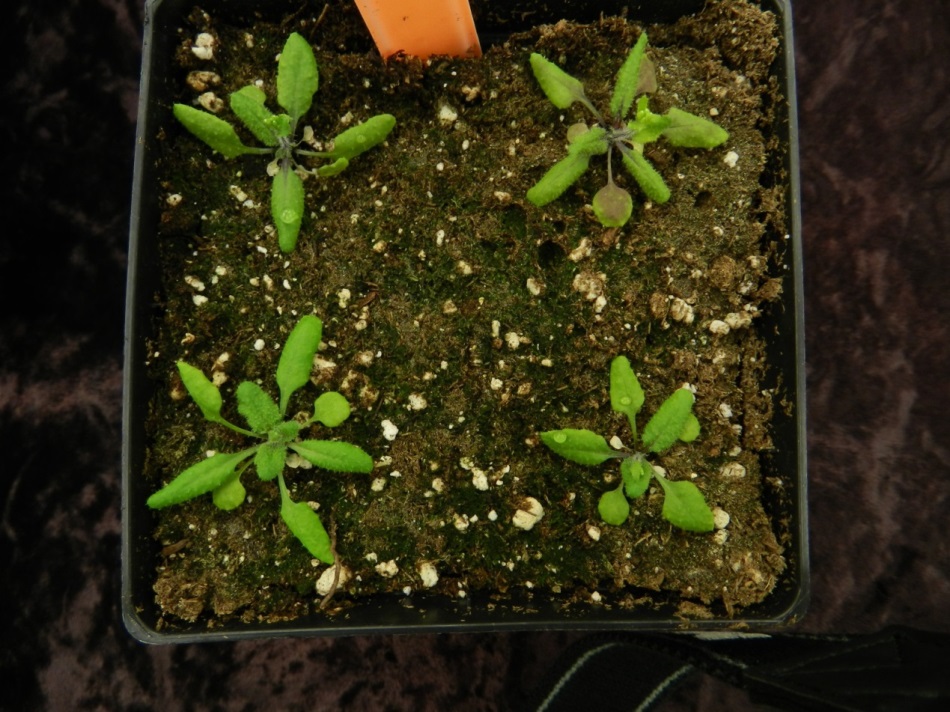

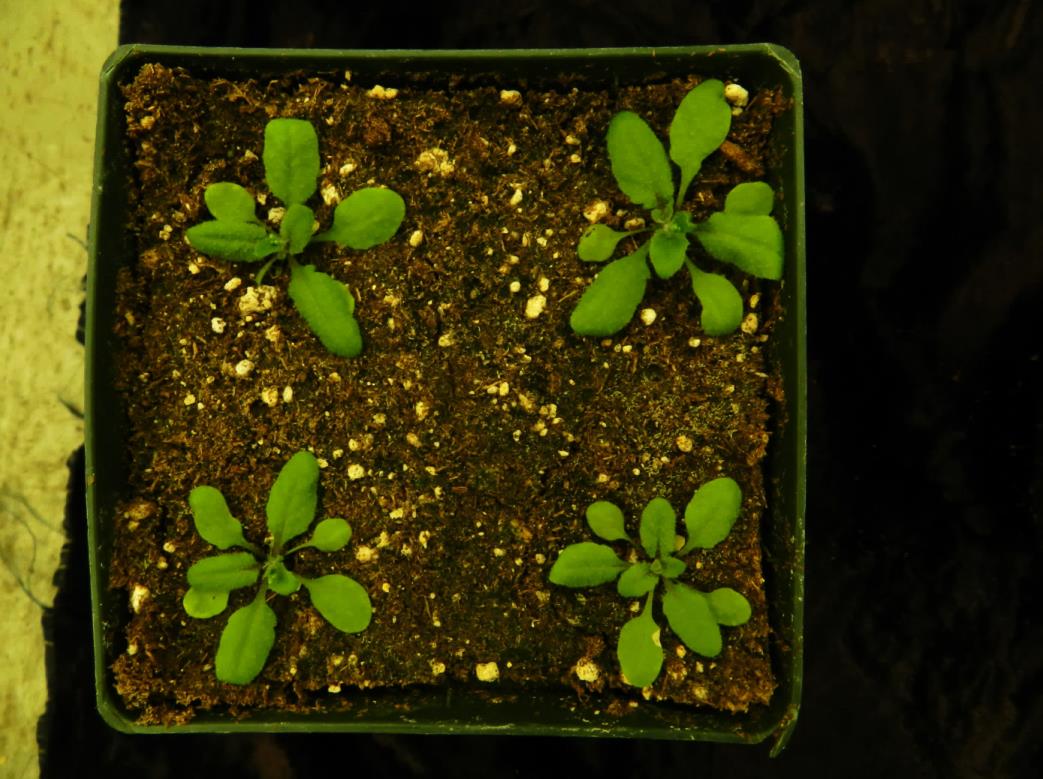

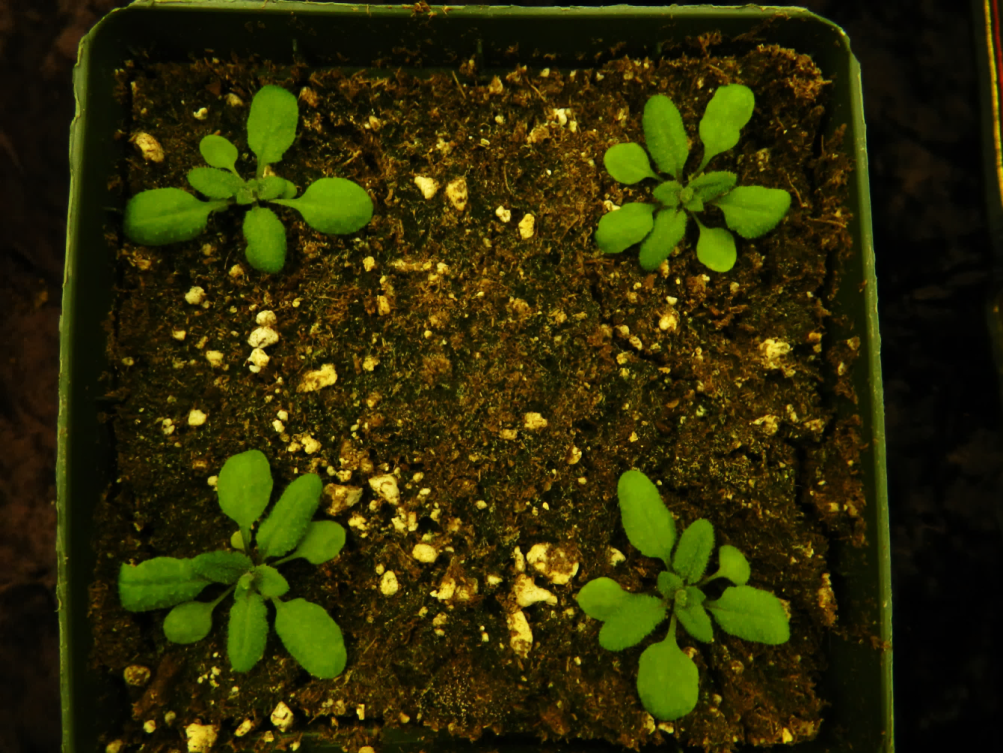


WT

SALK_067184

SALK_118027

(a)


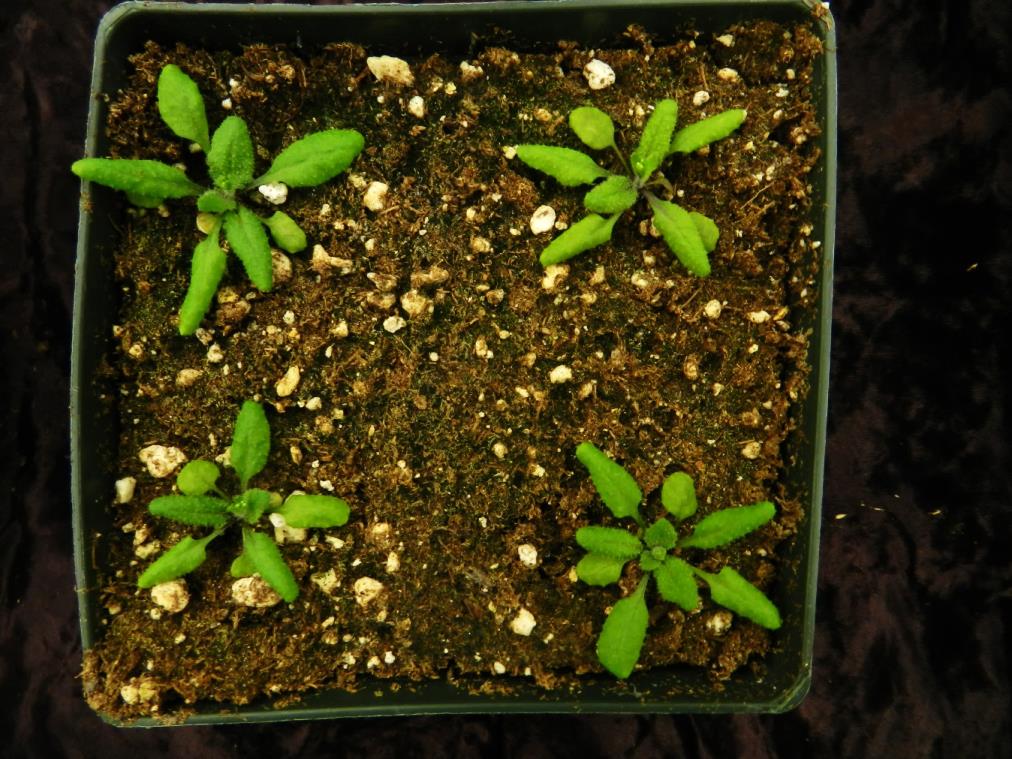


*atrtnlb3 atrtnlb6*

*atrtnlb3*

*atrtnlb6*

***

(b)

***

***

***

**Figure S4.** Characterization of T-DNA mutants. (a) Phenotype of the Arabidopsis ecotype Col-0 (WT) and homozygous T-DNA mutants *atrtnlb3*, *atrtnlb6*, and *atrtnlb3* *atrtnlb6* at 12 days under normal growth condition. Scale bars, 2 cm. (b) RT-qPCR assay on the mRNA expression level of *AtRTNLB3* and *AtRTNLB6* in the corresponding mutants.
