# Supplementary material for: Two plant membrane‐shaping reticulon‐like proteins play contrasting complex roles in turnip mosaic virus infection
Source: Mol Plant Pathol. 2024 Oct 16;25(10):e70017. doi: 10.1111/mpp.70017 (PMC11481689; doi:10.1111/mpp.70017)
Supplement: Supplementary file 5 — FIGURE S5. Molecular characterization of AtRTNLB3 and AtRTNLB6 overexpression plants. (a) Verification of overexpression of AtRTNLB3 or AtRTNLB6 in different transgenic Arabidopsis lines by western blot. CBB, Coomassie Brilliant Blue‐stained gel used as a loading control. (b) Phenotype of 14‐day‐old Arabidopsis wild‐type Col‐0 (WT) and the transgenic overexpression plants under normal growth condition. [file MPP-25-e70017-s001.docx]

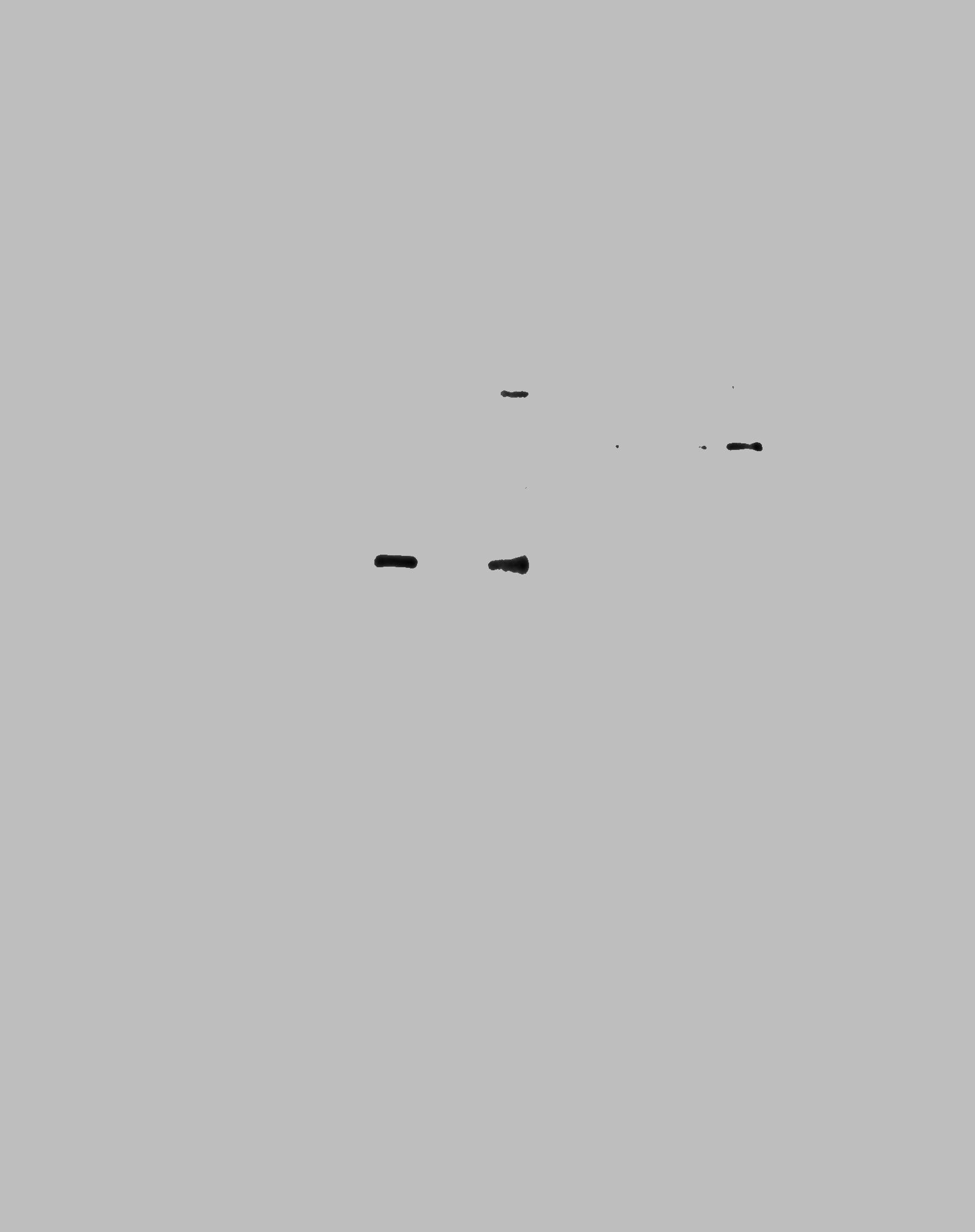


34

43

[kDa]

WT 1 2 3 4

OE lines for AtRTNLB6

CBB


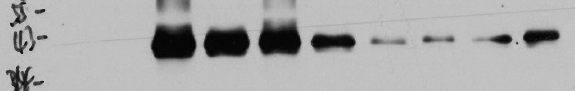


WT 1 2 3 4 5 6 7 8

OE lines for AtRTNLB3


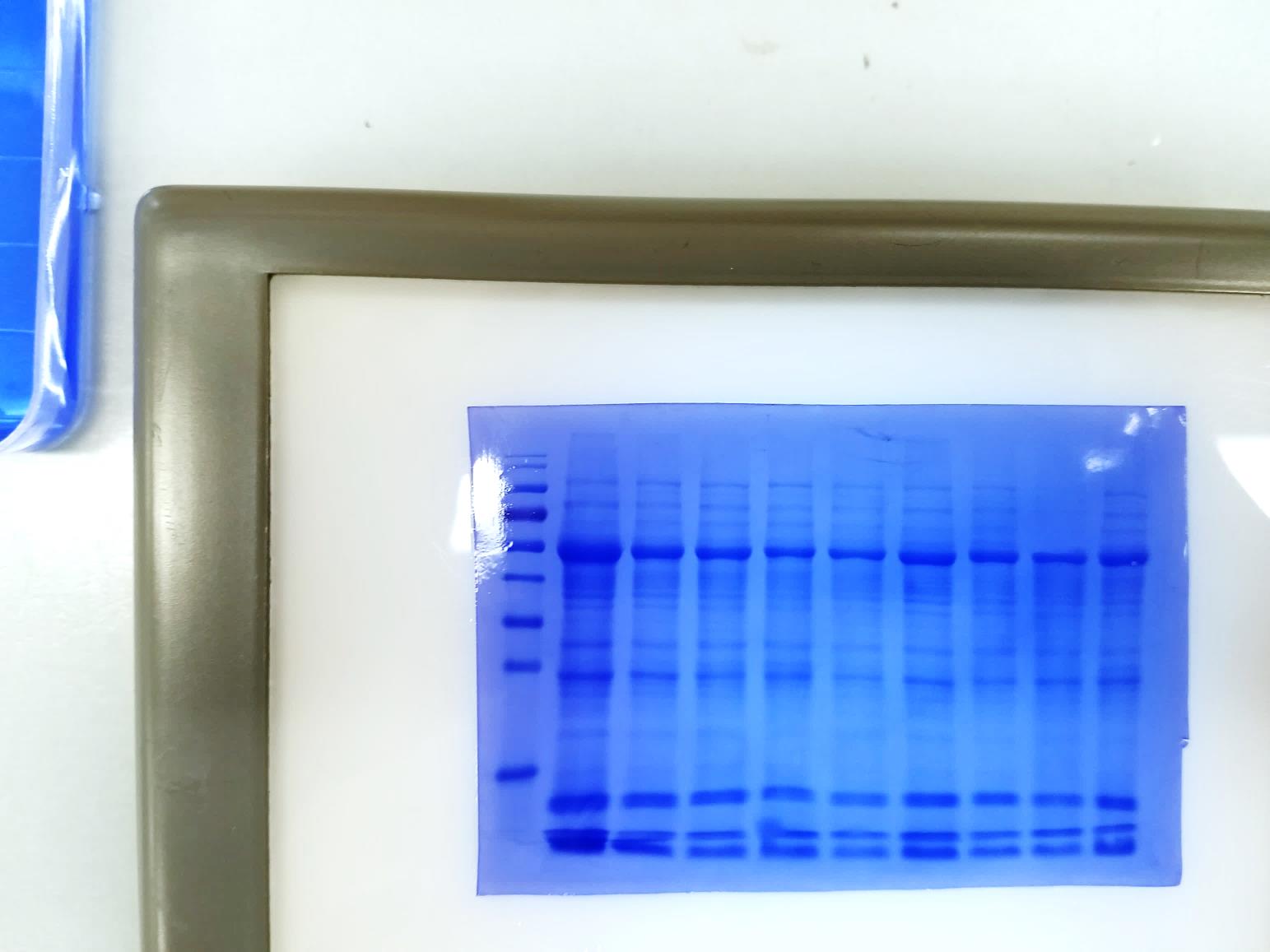

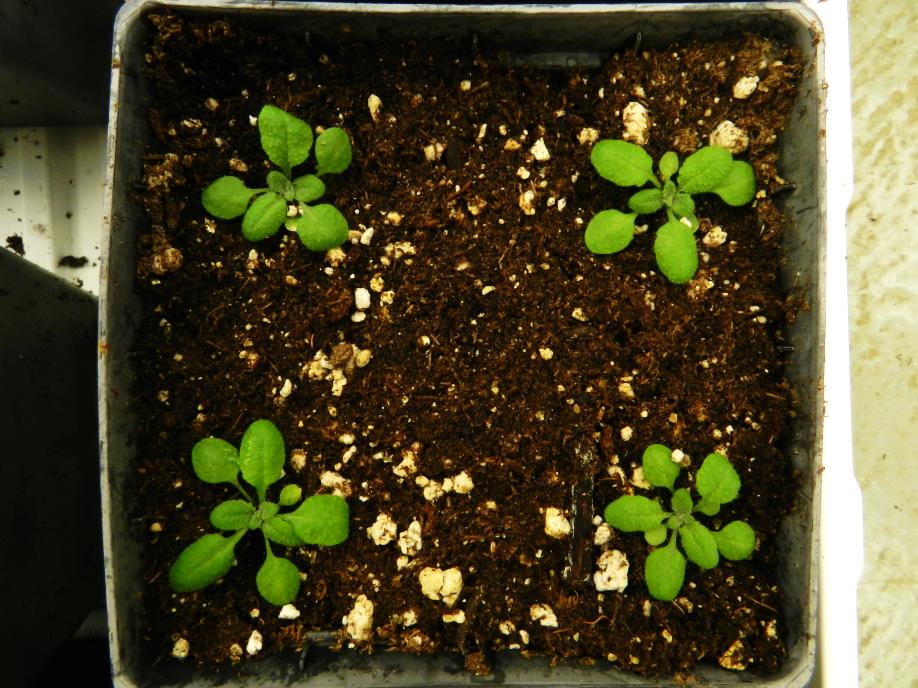

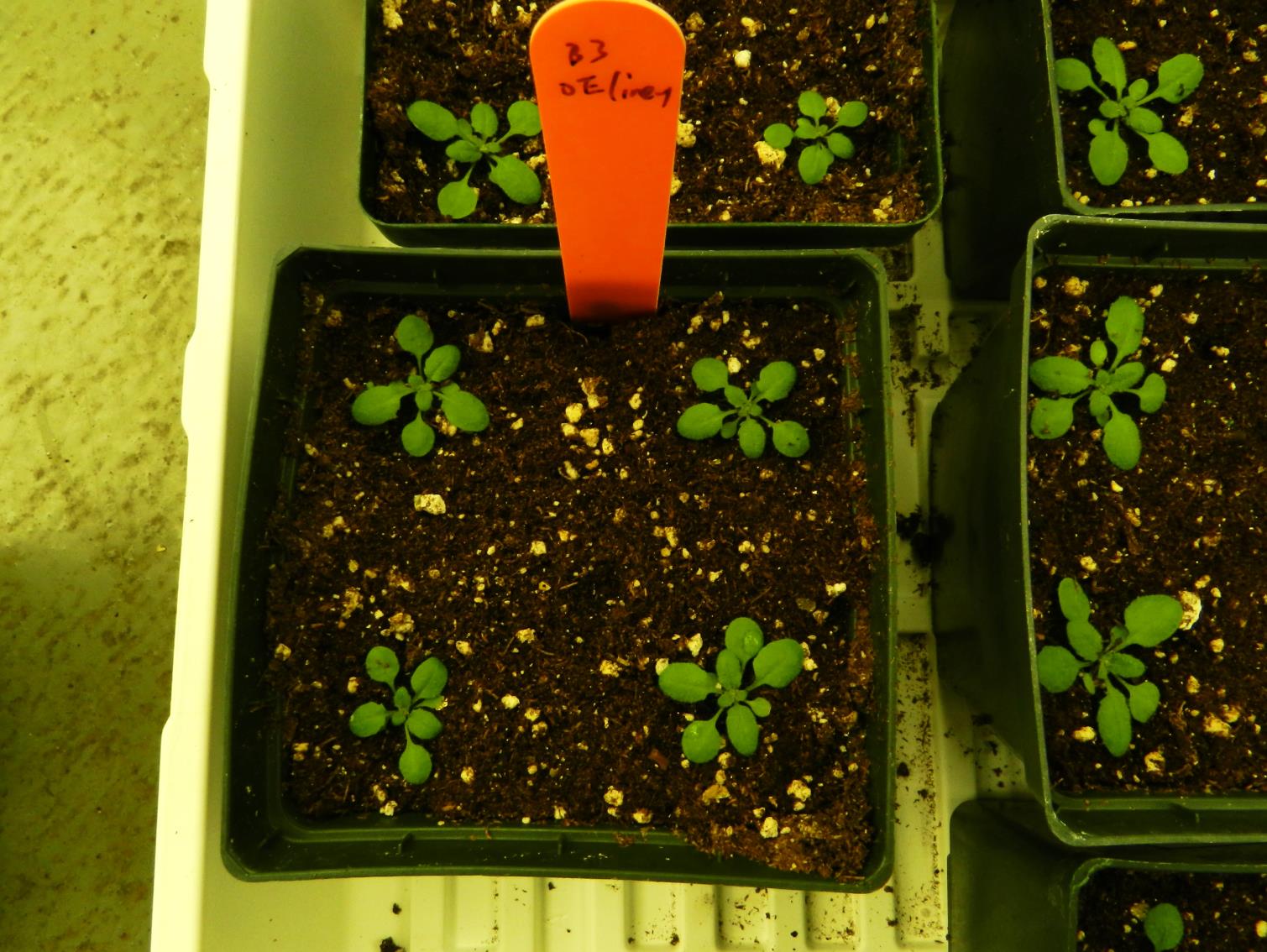

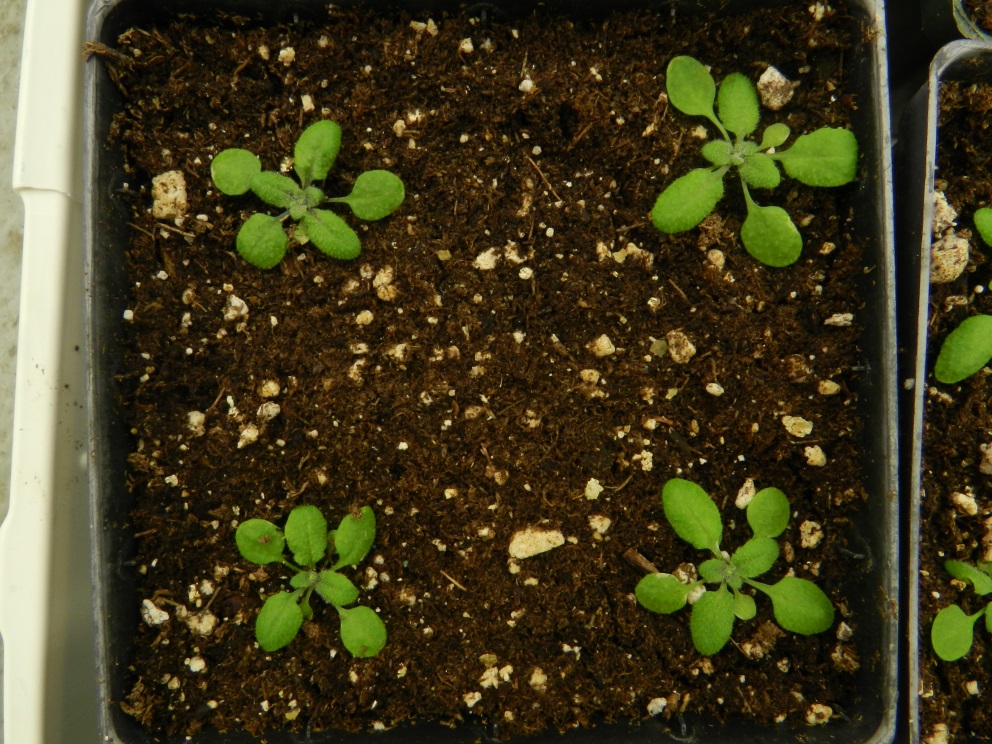

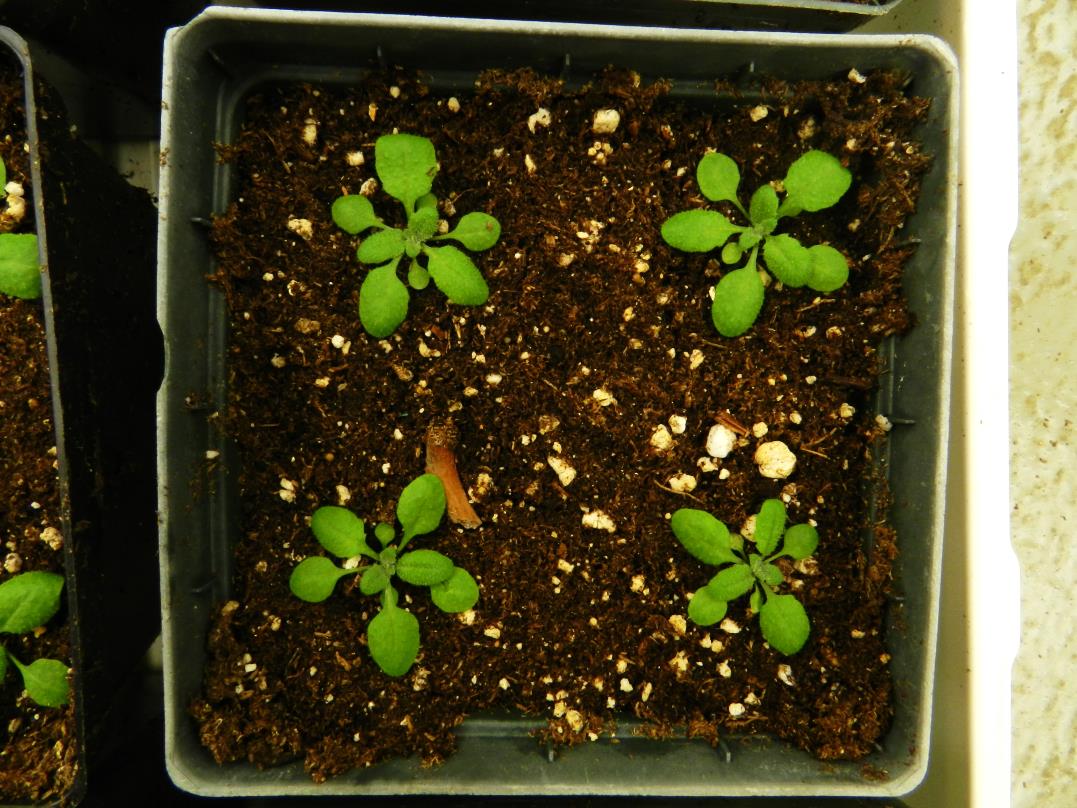

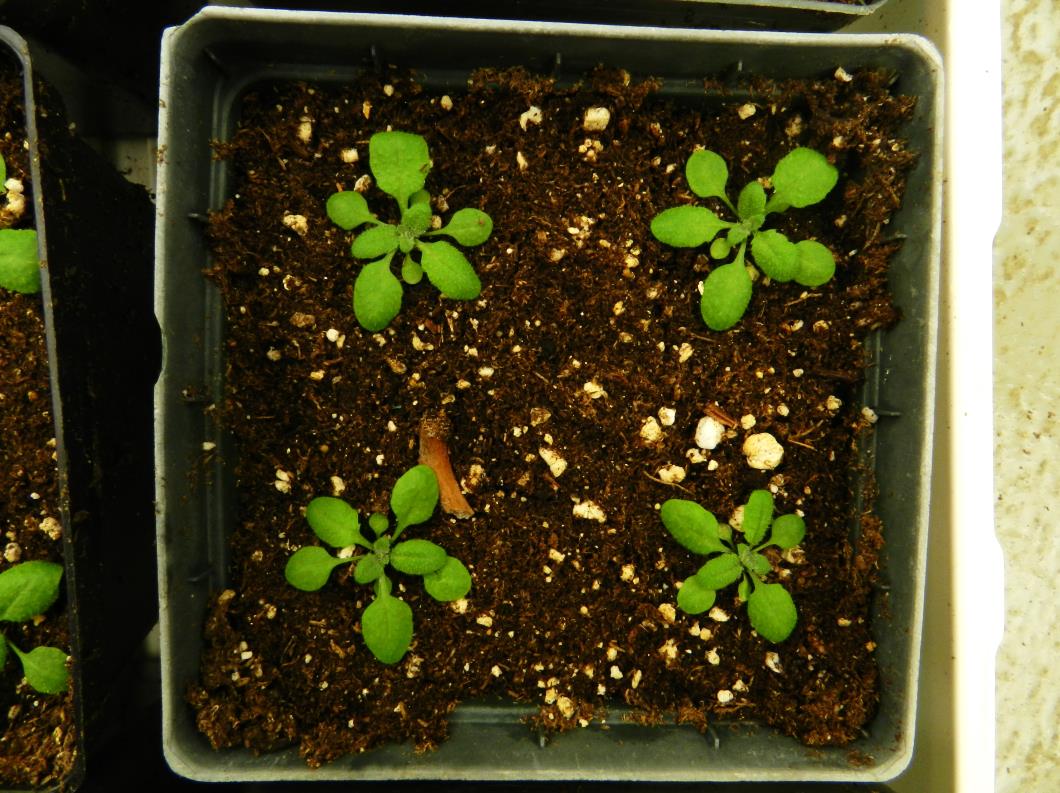


WT

35S:AtRTNLB3oe1

35S:AtRTNLB3oe2

35S:AtRTNLB6oe2

35S:AtRTNLB6oe4

(a)

(b)


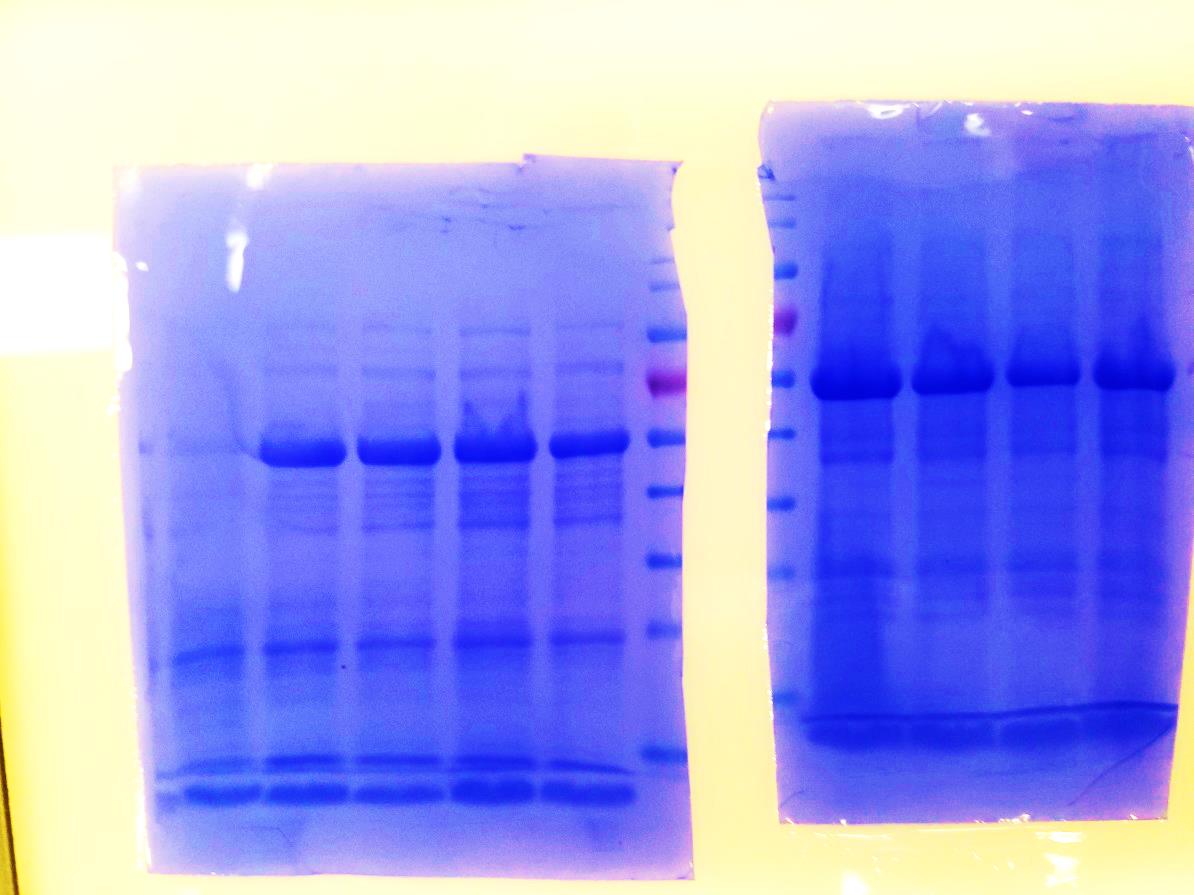


**Figure S5.** Molecular characterization of *AtRTNLB3* and *AtRTNLB6* overexpression plants. (a) Verification of overexpression of *AtRTNLB3* or *AtRTNLB6* in different transgenic Arabidopsis lines by Western blot. CBB, Coomassie brilliant blue-stained gel used as a loading control. (b) Phenotype of 14-days old Arabidopsis wild type Col-0 (WT) and the transgenic overexpression plants under normal growth condition.
