# Supplementary material for: Two plant membrane‐shaping reticulon‐like proteins play contrasting complex roles in turnip mosaic virus infection
Source: Mol Plant Pathol. 2024 Oct 16;25(10):e70017. doi: 10.1111/mpp.70017 (PMC11481689; doi:10.1111/mpp.70017)
Supplement: Supplementary file 6 — FIGURE S6. Multiple‐sequence alignments of the 6K2 protein sequences of 16 potyvirus members. The 6K2 sequences of different potyviruses were retrieved from the NCBI GenBank database under the GenBank accession numbers: Turnip mosaic virus (TuMV; EF028235.1), Sweet potato feathery mottle virus (SPFMV; AB465608.1), Tobacco vein banding mosaic virus (TVBMV; EU734432.1), Plum pox virus (PPV; NC_001445.1), Lily mottle virus (LMoV; AB570195.1), Bean yellow mosaic virus (BYMV; JX173278.1), Potato virus A (PVA; AJ131402.1), Papaya ringspot virus (PRSV; X97251.1), Chilli veinal mottle virus (ChiVWV; AJ972878.1), Tobacco etch virus (TEV; NC_001555.1), Potato virus Y (PVY; AJ439544.2), Watermelon mosaic virus (WMV; EU660581.1), Soybean mosaic virus (SMV; AF241739.1), Bean common mosaic virus (BCMV; HQ229995.1), Zucchini mosaic virus (ZYMV; AF014811.2) and Cowpea aphid‐borne mosaic virus (CABMV; NC004013.1). The alpha‐helix motif in 6K2 is indicated with red rectangle. [file MPP-25-e70017-s006.docx]

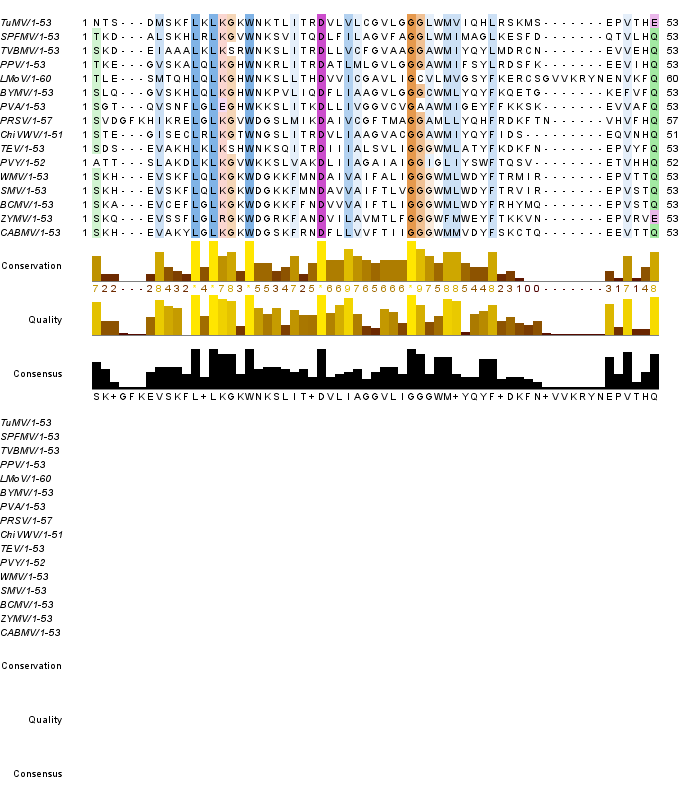


**Figure S6.** Multiple-sequence alignments of the 6K2 protein sequences of 16 potyvirus members. The 6K2 sequences of different potyviruses were retrieved from the NCBI GenBank database under the GenBank accession numbers: *Turnip mosaic virus* (TuMV; EF028235.1), *Sweet potato feathery mottle virus* (SPFMV; AB465608.1), *Tobacco vein banding mosaic virus* (TVBMV; EU734432.1), *Plum pox virus* (PPV; NC_001445.1), *Lily mottle virus* (LMoV; AB570195.1), *Bean yellow mosaic virus* (BYMV; JX173278.1), *Potato virus A* (PVA; AJ131402.1), *Papaya ringspot virus* (PRSV; X97251.1), *Chilli veinal mottle virus* (ChiVWV; AJ972878.1), *Tobacco etch virus* (TEV; NC_001555.1), *Potato virus Y* (PVY; AJ439544.2), *Watermelon mosaic virus* (WMV; EU660581.1), *Soybean mosaic virus* (SMV; AF241739.1), *Bean common mosaic virus* (BCMV; HQ229995.1), *Zucchini mosaic virus* (ZYMV; AF014811.2), and *Cowpea aphid-borne mosaic virus* (CABMV; NC004013.1). The alpha helix motif in 6K2 is indicated with red rectangle.
