# Supplementary material for: Two plant membrane‐shaping reticulon‐like proteins play contrasting complex roles in turnip mosaic virus infection
Source: Mol Plant Pathol. 2024 Oct 16;25(10):e70017. doi: 10.1111/mpp.70017 (PMC11481689; doi:10.1111/mpp.70017)
Supplement: Supplementary file 7 — FIGURE S7. Reverse transcription‐quantitative PCR detection of the viral CP RNA accumulation level in the upper new leaves of Nicotiana benthamiana agroinfiltrated with TuMV and different mutants at 28 days post‐agroinfiltration. F‐box was used as an internal control. Data represent means with SD of three biological replicates. NS, no significant difference. [file MPP-25-e70017-s009.docx]

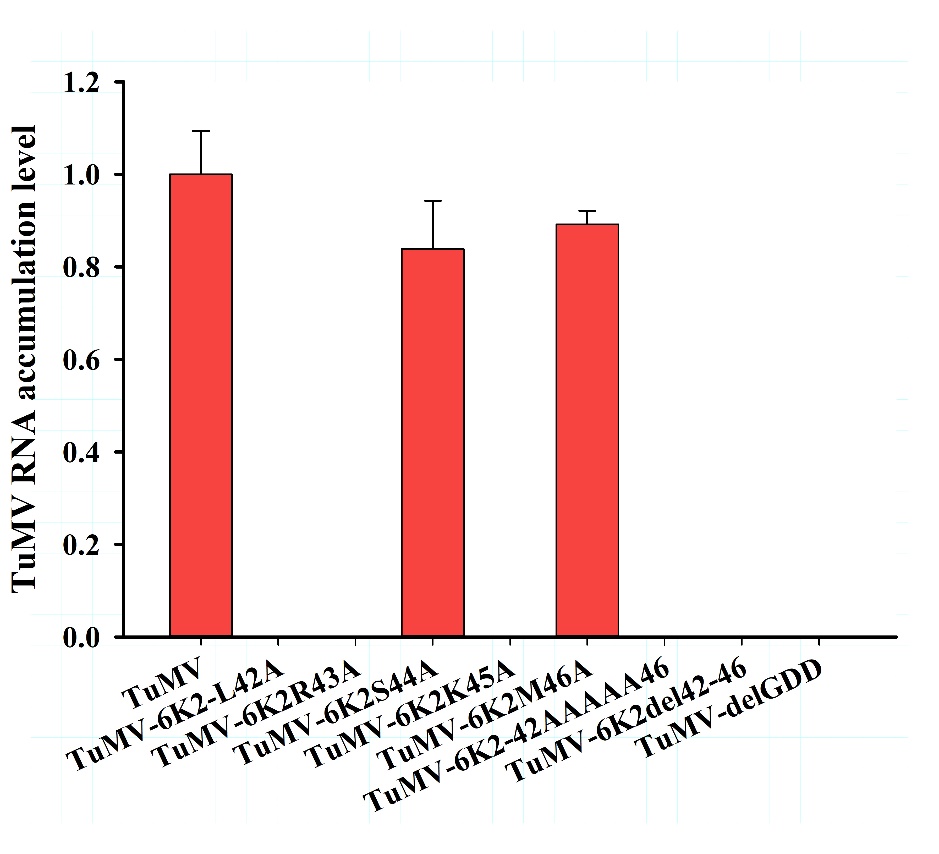


NS

NS

**Figure S7.** RT-qPCR detection of the viral cp RNA accumulation level in the upper new leaves of *N*. *benthamiana* agroinfiltrated with TuMV and different mutants at 28 days post agroinfiltration. *F-box* was used as an internal control. Data represent means with SD of three biological replicates. NS, no significant difference.
