# Supplementary material for: Two plant membrane‐shaping reticulon‐like proteins play contrasting complex roles in turnip mosaic virus infection
Source: Mol Plant Pathol. 2024 Oct 16;25(10):e70017. doi: 10.1111/mpp.70017 (PMC11481689; doi:10.1111/mpp.70017)
Supplement: Supplementary file 8 — FIGURE S8. Subcellular localization of 6K2‐GFP and its mutants 6K2‐del42‐46‐GFP or 6K2‐42AAAAA46‐GFP in Nicotiana benthamiana leaves. Chloroplast autofluorescence is shown in red. Scale bar = 20 μm. [file MPP-25-e70017-s005.docx]

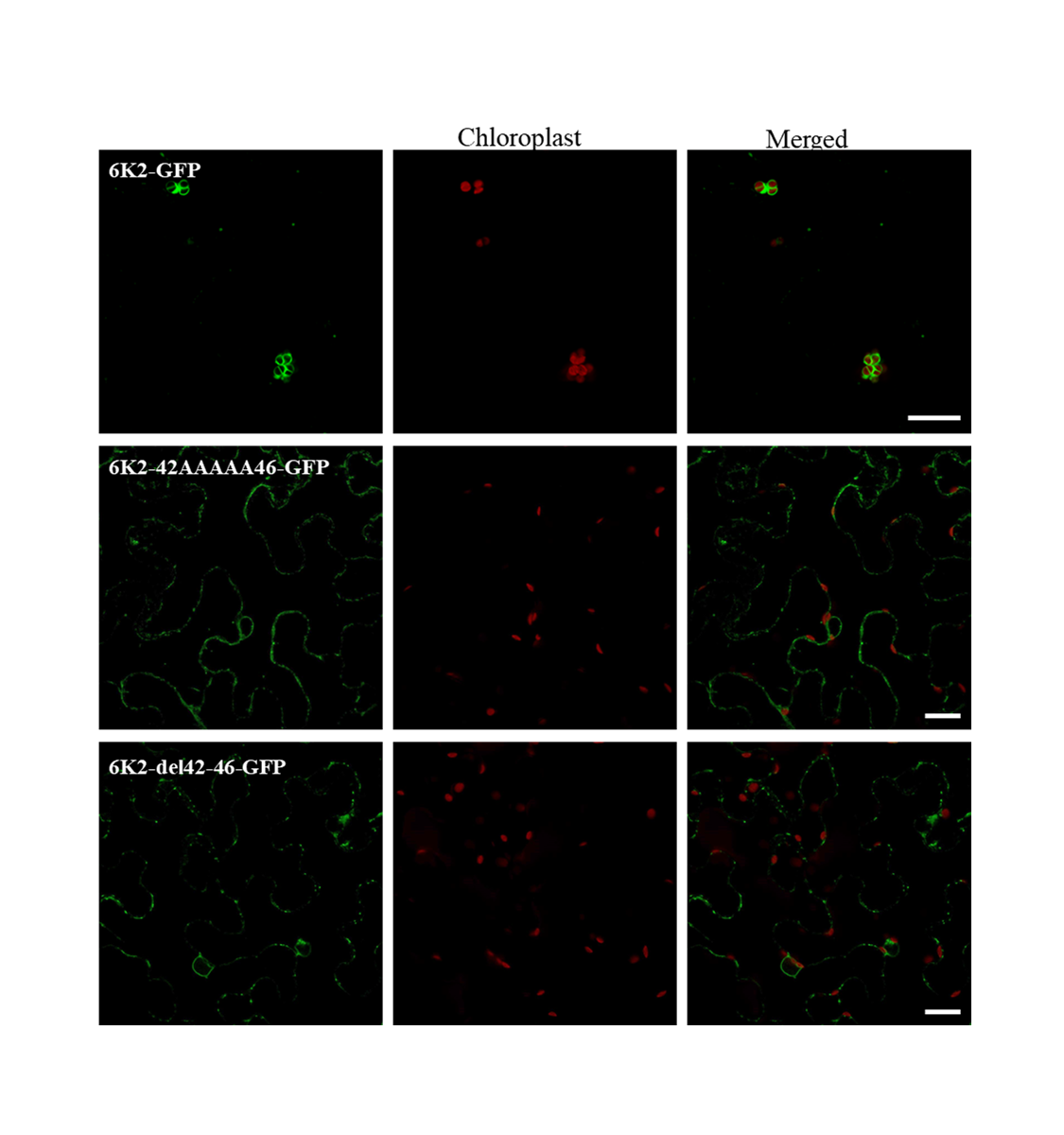


**Figure S8.** Subcellular localization of 6K2-GFP and its mutants 6K2-del42-46-GFP or 6K2-42AAAAA46-GFP in *N. benthamiana* leaves. Expression vectors were agroinfiltrated into *N. benthamiana* leaf tissues. The infiltrated area was visualized under a confocal microscope. Chloroplast autofluorescence is shown in red. Images were taken at 48 hpi. Scale bar = 20 um.
