# Supplementary material for: Two plant membrane‐shaping reticulon‐like proteins play contrasting complex roles in turnip mosaic virus infection
Source: Mol Plant Pathol. 2024 Oct 16;25(10):e70017. doi: 10.1111/mpp.70017 (PMC11481689; doi:10.1111/mpp.70017)
Supplement: Supplementary file 9 — TABLE S1. List of primers used in this study. [file MPP-25-e70017-s003.docx]

**Table S1.** List of primers used in this study

| **Primer names** | **Sequences (5’ to 3’)** | **Notes** |
| --- | --- | --- |
| BP-AtRTNLB3-F | GGGGACAAGTTTGTACAAAAAAGCAGGCTTCatggcggaagagcacaagca | Primers are designed for cloning into the entry vector pDONR221.  GenBank Accession no.: NM_001198378.2 |
| BP-AtRTNLB3-R | GGGGACCACTTTGTACAAGAAAGCTGGGTCatctttcttcttgataaagg |  |
| BP-AtRTNLB6-F | GGGGACAAGTTTGTACAAAAAAGCAGGCTTCATGGCGGAAGAATTGGAGAA | Primers are designed for cloning into the entry vector pDONR221.  GenBank Accession no.: NM_116021.4 |
| BP-AtRTNLB6-R | GGGGACCACTTTGTACAAGAAAGCTGGGTCACCCAACTTTGCCTTAAGGG |  |
| BP-NbRTNLB2-F | GGGGACAAGTTTGTACAAAAAAGCAGGCT TCATGGCCGATCACGCCGGCGAGCACG | Niben101Scf28925g00001.1 and Niben101Scf01756g02004.1.  https://solgenomics.net/jbrowse_solgenomics/?data=data%2Fjson%2FNiben1.0.1&loc=Niben101Scf01756%3A325401..331561&tracks=Nibenv101_gene_models%2CDNA&highlight= |
| BP-NbRTNLB2-R | GGGGACCACTTTGTACAAGAAAGCTGGGTC TAGGAACTTTTTGTCTTTCAGTGG |  |
| BP-NbRTNLB5-F | GGGGACAAGTTTGTACAAAAAAGCAGGCT TCATGGCAGAACACGTTGAGAATTCGG |  |
| BP-NbRTNLB5-R | GGGGACCACTTTGTACAAGAAAGCTGGGTCTTCCCTCTTCTTGTCTTTCAATGG |  |
| BP-AtRTNLB3-N-R | GGGGACCACTTTGTACAAGAAAGCTGGGTCactagcactagaccacaaga | Primers are designed for interaction domain mapping, and cloning into the entry vector pDONR221. |
| BP-AtRTBLB3-M-F | GGGGACAAGTTTGTACAAAAAAGCAGGCTTCaccttcattcacaagtcacc |  |
| BP-AtRTBLB3-M-R | GGGGACCACTTTGTACAAGAAAGCTGGGTCgctaccaactttggacaaaa |  |
| BP-AtRTNLB3-C-F | GGGGACAAGTTTGTACAAAAAAGCAGGCTTCtcctgcaacttcttgacctt |  |
| BP-AtRTNLB6-N-R | GGGGACCACTTTGTACAAGAAAGCTGGGTCATGATACTCAACCAATTCAA |  |
| BP-AtRTBLB6-M-F | GGGGACAAGTTTGTACAAAAAAGCAGGCTTCTTCTTGAGTCTTGTGTGTCA |  |
| BP-AtRTBLB6-M-R | GGGGACCACTTTGTACAAGAAAGCTGGGTCGTCCCTTCCTAAGGCAATGCT |  |
| BP-AtRTNLB6-C-F | GGGGACAAGTTTGTACAAAAAAGCAGGCTTCTTGAAGAAATTTCTCATGG |  |
| atrtnlb3-LP (SALK_067184C) | GCGAGAGCAAGAATTGAAATG | Primers were designed on the T-DNA Primer Design server (http://signal.salk.edu/tdnaprimers.html). |
| atrtnlb3-RP (SALK_067184C) | CCTCAAGTTTCTTATTGGCCC |  |
| atrtnlb6-LP (Salk_118027) | TTTGTCCCTCCACAAGAACAC |  |
| atrtnlb6-RP (Salk_118027) | AATTGTCGGAGATGAATGTCTC |  |
| LBb1.3 | ATTTTGCCGATTTCGGAAC |  |
| qPCR-TuMV CP-F | TGGCTGATTACGAACTGACG | Primers are designed based on the submitted NCBI sequence for the TuMV isolate (EF028235.1). |
| qPCR-TuMV CP-R | CTGCCTAAATGTGGGTTTGG |  |
| qPCR-AtF-box-F | GGCTGAGAGGTTCGAGTGTT | Inter control gene for qRT-PCR in Arabidopsis and *N. benthamiana*, respectively. |
| qPCR-AtF-box-R | GGCTGTTGCATGACTGAAGA |  |
| qPCR-NbF-box-F | GGCACTCACAAACGTCTATTTC |  |
| qPCR- NbF-box-R | ACCTGGGAGGCATCCTGCTTAT |  |
| qPCR-AtRTNLB3-F | TATTAATTCCCAACGGCCAA |  |
| qPCR-AtRTNLB3-R | TCAGTAAGCGATGAGCGAGA |  |
| qPCR-AtRTNLB6-F | CTGATGGAGAAGATCGCTGA |  |
| qPCR-AtRTNLB6-R | AGCTGACGGAGACTCTGGTT |  |
| qPCR-AtPDLP1-F | TTTCAGTGCCGTGGCGATCT | GenBank Accession no.: NM_123765.3 |
| qPCR-AtPDLP1-R | TCCAGCGAGGTGAACACGAG |  |
| qPCR-AtPDLP5-F | GTCACCTCAACCGCCCAAAC | GenBank Accession no.: NM_105737.4 |
| qPCR-AtPDLP5-R | CGCTTGATACGCACGTCGAG |  |
| qPCR-AtPR1-F | CACATCCGAGTCTCACTGAC | SA pathway-related gene  Lim et al. 2016, Cell Host&Microbe |
| qPCR-AtPR1-R | CAGACTCATACACTCTGGTG |  |
| qPCR-AtIRE1b-F | TGGGCGGTGGGATGAGAAAC | GenBank Accession no.: NM_122344.5 |
| qPCR-AtIRE1b-R | CCCGTAACAAATCGCGGATGC |  |
| BP-TuMV-6K2-F | GGGGACAAGTTTGTACAAAAAAGCAGGCTTCatgaacaccagcgacatgagcaaattc |  |
| BP-TuMV-6K2-R | GGGGACCACTTTGTACAAGAAAGCTGGGTCttcatgggttacgggttcggac |  |
| BP-TuMV-6K2ΔN1-20-F | GGGGACAAGTTTGTACAAAAAAGCAGGCTTCatgacgcgagatgtgctggtactttg |  |
| BP-TuMV-6K2ΔC42-53-R | GGGGACCACTTTGTACAAGAAAGCTGGGTCgtgctgaataaccatccacaatc |  |
| BP-TuMV-6K2ΔC47-53-R | GGGGACCACTTTGTACAAGAAAGCTGGGTCcatctttgaccgcaggtgctg |  |
| BP-6K2L42A-R | GGGGACCACTTTGTACAAGAAAGCTGGGTCttcatgggttacgggttcggacatctttgaccgAGCgtgctgaataaccatccacaatccac |  |
| BP-6K2R43A-R | GGGGACCACTTTGTACAAGAAAGCTGGGTCttcatgggttacgggttcggacatctttgaCGCcaggtgctgaataaccatccacaatccac |  |
| BP-6K2S44A-R | GGGGACCACTTTGTACAAGAAAGCTGGGTCttcatgggttacgggttcggacatcttTGCccgcaggtgctgaataaccatccacaatccac |  |
| BP-6K2K45A-R | GGGGACCACTTTGTACAAGAAAGCTGGGTCttcatgggttacgggttcggacatAGCtgaccgcaggtgctgaataaccatccacaatccac |  |
| BP-6K2M46A-R | GGGGACCACTTTGTACAAGAAAGCTGGGTCttcatgggttacgggttcggaAGCctttgaccgcaggtgctgaataaccatccacaatccac |  |
| BP-6K2-42AAAAA46-R | GGGGACCACTTTGTACAAGAAAGCTGGGTCttcatgggttacgggttcggaGGCAGCAGCAGCAGCgtgctgaataaccatccacaatccac |  |
| BP-6K2Δ42-46-R | GGGGACCACTTTGTACAAGAAAGCTGGGTCttcatgggttacgggttcggagtgctgaataaccatccacaatccac |  |
| P3-SnabI-F | gattacgtagtagtatgtcttggactagtag |  |
| NIb-Mlu I | ggacgcgtagaacttgcgtatctcgtgagttaac |  |
| 6K2Δ42-46-F | gattgtggatggttattcagcactccgaacccgtaacccatgaagcgaaag |  |
| 6K2Δ42-46-R | cttcatgggttacgggttcggagtgctgaataaccatccacaatccacc |  |
| 6K2-L42A-F | gtggattgtggatggttattcagcacgctcggtcaaagatgtccgaacccgtaacccatgaa |  |
| 6K2-L42A-R | ttcatgggttacgggttcggacatctttgaccgAGCgtgctgaataaccatccacaatccac |  |
| 6K2-R43A-F | gtggattgtggatggttattcagcacctggcgtcaaagatgtccgaacccgtaacccatgaa |  |
| 6K2-R43A-R | ttcatgggttacgggttcggacatctttgaCGCcaggtgctgaataaccatccacaatccac |  |
| 6K2-S44A-F | gtggattgtggatggttattcagcacctgcgggcaaagatgtccgaacccgtaacccatgaa |  |
| 6K2-S44A-R | ttcatgggttacgggttcggacatcttTGCccgcaggtgctgaataaccatccacaatccac |  |
| 6K2-K45A-F | gtggattgtggatggttattcagcacctgcggtcagctatgtccgaacccgtaacccatgaa |  |
| 6K2-K45A-R | ttcatgggttacgggttcggacatAGCtgaccgcaggtgctgaataaccatccacaatccac |  |
| 6K2-M46A-F | gtggattgtggatggttattcagcacctgcggtcaaaggcttccgaacccgtaacccatgaa |  |
| 6K2-M46A-R | ttcatgggttacgggttcggaAGCctttgaccgcaggtgctgaataaccatccacaatccac |  |
| 6K2-42AAAAA46-F | gtggattgtggatggttattcagcacGCTGCTGCTGCTGCCtccgaacccgtaacccatgaa |  |
| 6K2-42AAAAA46-R | ttcatgggttacgggttcggaGGCAGCAGCAGCAGCgtgctgaataaccatccacaatccac |  |
